# Eye-tracking-based experimental paradigm to assess social-emotional abilities in young individuals with profound intellectual and multiple disabilities

**DOI:** 10.1371/journal.pone.0266176

**Published:** 2022-04-14

**Authors:** Thalia Cavadini, Yannick Courbois, Edouard Gentaz

**Affiliations:** 1 Department of Psychology, University of Geneva, Geneva, Switzerland; 2 ULR 4072 - PSITEC - Psychologie: Interactions Temps Émotions Cognition, Univ. Lille, Lille, France; 3 Swiss Center for Affective Sciences, University of Geneva, Geneva, Switzerland; 4 CNRS, Paris, France; University of Tübingen, GERMANY

## Abstract

Individuals with Profound Intellectual and Multiple Disabilities (PIMD) experience a combination of severe cognitive and motor impairments frequently associated with additional sensory deficits and numerous medical disorders. The purpose of the present study was to propose an experimental paradigm based on eye-tracking that combines various pre-existing tasks from infancy research as an assessment tool. This would enable the investigation of social-emotional abilities in nine young individuals with PIMD through their visual preferences for different types of stimuli. The first objective was to test the feasibility of this paradigm, by expecting individuals to look more at the tasks’ presentation screen than elsewhere during its implementation. The second objective was to investigate whether PIMD individuals exhibit visual preferences for (a) biological (vs. non-biological) motion, (b) socially salient (vs. non-social) scenes, (c) the facial area of the eyes (vs. the mouth), (d) happy (vs. angry) faces, (e) objects of joint attention (vs. non-looked at ones), and for (f) prosocial (vs. anti-social) behaviors similar to those of a control group of typically developing children aged two years on average. Overall, the feasibility of this paradigm proved to be good, resulting in high individual looking rates that were not affected by the presentation or the content of the tasks. Analyses of individual social-emotional abilities, supported by the visual preference patterns of each PIMD individual, firstly revealed strong—but expected—variability both within and between subjects, and secondly highlighted some individual task-specific abilities although few similarities between these individual results and those of the control group were found. These findings underline the great relevance of using this type of paradigm for assessing PIMD individuals and thus contribute to a better understanding of their social and emotional development.

## Introduction

The clinical population with Profound Intellectual and Multiple Disabilities (PIMD) is described by Nakken and Vlaskamp (2002; 2007) as a heterogeneous group of individuals with severe to profound cognitive disabilities (with a statistically estimated IQ below 20–25, and an overall developmental age of 24 months or less) and extensive motor limitations [1, 2] Additional sensory impairments are also frequently experienced by PIMD individuals [3] They also have an overall risk of developing numerous health conditions or medical complications, and almost all require regularly administered medication [4]. This population forms a physically vulnerable group of persons with a heavy or total dependence on support from others for everyday tasks and daily living activities [5]. In order to provide appropriate personalized assistance to people with such severe disabilities, support persons have to be sufficiently knowledgeable about individual needs, possibilities, or preferences [6]. This is particularly difficult to do with the PIMD population since assessing persons with PIMD is a challenging process with multiple issues [2, 5].

The extreme heterogeneity that characterizes the population with PIMD is the first limitation to proceed with their psychological evaluation of competences. However, according to Nakken and Vlaskamp (2007), assessing a person with PIMD is also extremely difficult because each individual represents a unique configuration of abilities and constraints to functioning, as well as an idiosyncratic communicative repertoire [2]. Due to the extensive problems in communication they experience (e.g., limited or non-existent use of expressive nor receptive language) coupled with the fact that most PIMD individuals are unable to reach, point, or grasp objects, they are only able to express themselves through an extremely limited range of communicative signals [7]. Therefore, the use of common standardized tests or self-report questionnaires with these individuals is invalidated [8, 9]. In addition, the severity and multiplicity of disabilities in the PIMD population result in unconventional reactions and inconsistent responses to sensory stimuli of items from common sensorimotor assessment batteries [10, 11]. Given these limitations in assessing individuals with PIMD, observation remains the most effective method today [5, 12]. Facilitating the report of observed behaviors that are not language-based [13], a number of indirect observational scales are currently available, especially for support persons willing to gather key-information from these individuals in order to provide appropriate assistance. However, the psychometric properties of such tools are generally limited [5]. Indeed, observation-based methods involve examining individual behaviors and interpreting them according to the knowledge of the evaluator (e.g., a professional or a family member) about the person being assessed, whereas it has been shown that preference rankings based on respondents’ opinions do not consistently coincide with the results of formal assessment procedures consisting of direct observation of both approach and avoidance behaviors [14–16]. Therefore, there is a need to improve the evaluation process of individuals with PIMD by diversifying and multiplying the instruments (given the extensive inter-individual variability characterizing this population) and by conducting direct evaluations for a deeper investigation of individual competences. This would contribute to better understand the psychological development of persons with PIMD who are frequently labelled as ‘untestable’ because of the theoretical and practical difficulties involved in this process [2, 17, 18].

Among the extremely limited behavioral responses available to PIMD individuals, oculomotor activity is generally minimally impacted (at least in the absence of cerebral visual impairment [19] by the severe and multiple disabilities that characterize this population [20, 21]. As oculomotor activity is considered to be an appropriate measure to use when other response systems are limited [22], it therefore appears to be a highly relevant measure in the assessment process. This is especially true in the framework of a visual preference procedure, which is probably one of the most suitable methods available considering the short time-window of alertness of the PIMD population [23].

The present study offers an experimental paradigm using a classic fixed-trial visual preference procedure initially designed by Fantz (1963) for testing visual responsiveness to various object features in newborn infants [24]. The measure of interest is commonly the looking duration at each stimulus or the percentage of looking duration at one or the other stimulus, to determine whether there is a statistically significant difference in the time infants spend looking at each of two paired stimuli, and thus conclude a visual preference for one of them, indicating that the subject discriminates between the two stimuli. Although this type of paradigm has been widely used to study the development of early abilities in infancy over the last 50 years, very little work has been achieved to date using gaze orientation in persons with PIMD in order to explore their psychological development. An exception is Chard, Roulin and Bouvard (2014) who have recently attempted using a visual habituation procedure [25]. In this study, the authors repeatedly exposed 15 adults with PIMD to two different objects to test the existence of the habituation/novelty reaction phenomenon in these individuals following the principles of a participant-controlled procedure. By conducting both qualitative and quantitative analyses of the observed behaviors (e.g., attention getting and holding processes) using a specially designed apparatus, they were able to show that the PIMD participants presented the same habituation profiles generally obtained in infancy research [25].

Based on the hypothesis that a gaze fixation on a specific point provides a measure of attention [26], eye-tracking technology has been widely used for the study of visual attention to various stimuli types. In addition, the use of eye-tracking technology as an experimental measurement provides great spatial and temporal precision: by defining Areas of Interest (AOIs) on the stimuli presented on the screen, this device allows the precise examination of individual visual exploration (e.g., time spent looking at the *eyes* vs. at the *mouth* of a face) [27]. Thus, a growing number of studies have used this technology to investigate various abilities in typically developing (TD) infants [28] and in older children [29].

This technology has also proven its relevance in disability research, particularly in the field of Autism spectrum disorders (ASD) [30]. Considering the relevance of this technology in the early identification of risk factors for autism [31, 32] and given that the feasibility of eye-tracking-based assessments in low-resource settings has been demonstrated [33], a recent study has even developed stimuli specifically adapted to the South American population in order to provide a tool that can help local practitioners to identify the early signs of autism in this geographic area [34]. Some authors have also highlighted the great relevance of using this technology in computer-based support programs, in particular by serving as an assistive device [35], especially to support the communication of individuals with limited general body movement [36].

In order to better understand the psychological development of individuals with PIMD, the present study intended to perform an eye-tracking-based experimental paradigm using a visual preference procedure for assessing six social-emotional abilities. For this purpose, we designed a paradigm combining different pre-existing tasks from infancy research whose experimental contents have provided significant contributions to current scientific knowledge on the typical development of early social communication.

Starting with [1] *the discrimination of biological vs*. *non-biological motion*, likely one of the earliest perceptual abilities involved in the development of nonverbal communication [37] since it has been observed from birth in TD newborns [38], we were interested in determining whether PIMD individuals would show a preference for biological motion represented by dynamic point-light depicting human actions over random non-biological moving dots, through an adaptation of Johansson’s (1973) “Point-light” stimuli [39].

As a continuation of the previous ability, we then focused on [2] *social orienting* (SO), referring to the intrinsic ability to preferentially orient to our surrounding social environment [40], which autism research proved to be of paramount importance for the early development of social communication [41] since the lack of social orienting is thought to impair the social communicative development of children with ASD [42]. Using Franchini et al.’s (2016) stimuli [32], we wanted to investigate whether this preference for socially salient scenes would also be found in individuals with PIMD, by presenting not only basic white moving dots as in the previous task, but also concrete stimuli consisting in children dancing solo (vs. dynamic geometric motion).

Taking a closer look at concrete human stimuli, we then focused on human faces, which are known to be a highly salient stimulus type [43], whose visual processing and comprehension play a fundamental role in social communication [44]. Using pairs of emotional faces, we were interested in [3] *the visual scanning of facial features* by investigating whether the early typical exploration pattern of preferring the eye area [45] would also be exhibited by PIMD individuals, as well as [4] *the discrimination of emotions* and more specifically whether these individuals would be able to differentiate between happy and angry faces, two contrasting emotions that TD infants are able to discriminate as early as 7 months of age showing a preference for happy faces [46].

Beyond the visual exploration of static faces, we were then interested in [5] *the joint attention capacities* of individuals with PIMD. Referring to a co-created outcome of an interaction in which two people’s attention is directed towards each other or towards an object, joint attention requires both expressive (i.e., initiation of joint attention behaviors) and receptive (i.e., responding to joint attention behaviors; abbreviated here as RJA) abilities [47]. In the present study, we focused on RJA behaviors (i.e., the ability to follow the direction of the gaze and gestures of others to share a common point of reference [48]) of PIMD individuals by presenting them with videos created by Franchini et al. (2017) in which an actress directed attention to a moving object by looking at it with an intensely surprised expression [49].

Finally, after directing one’s gaze to what catches others’ attention, we then investigated whether PIMD individuals would be able to [6] *produce Socio-Moral Evaluations* (SME), i.e., the ability to understand others’ intentions by observing their actions and judging the moral valence of their behavior, which is fundamental to processing the social world and interactions [50]. We therefore focused on the SME abilities of PIMD individuals using an adaptation of Hamlin, Wynn, and Bloom’s (2007) “Climbing the hill” paradigm [51] through which they demonstrated that, as early as 6 months of age, TD infants were able to exhibit such evaluations by showing a visual preference for the agent performing a prosocial action (a helper aiding the climber) over an antisocial one (a hinderer pushing the climber down). Since PIMD individuals experience significantly reduced social interactions in their lives [52], it is particularly relevant to investigate whether they would exhibit similar visual preferences.

While the typical development of these six abilities in the first year of life has been widely studied to date, little work has already explored them in older infants, especially around the age of two years, which corresponds to the estimated developmental level of the PIMD population [1, 2, 53]. For this reason, we decided to also implement our paradigm with a group of TD children of this age, providing a baseline for case-control comparisons to better understand patterns of visual preference of each PIMD individual.

In summary, the first objective of the present study was to test the feasibility of the proposed eye-tracking-based experimental paradigm. We expected that participants would spend more than half of the session looking at the presentation screen (at least 50% of looking time) and that these looking times would not be influenced by effects related to the presentation (order and fatigue effects) or the content (visual stimuli) of the tasks. The second objective was to explore the social-emotional abilities of nine PIMD individuals (single-case analyses), by testing whether they showed expected visual preferences in six tasks, and by comparing their individual results to those of the control group.

## Methods

### Participants

The present study included two distinct populations: an average 2-year-old control group and young individuals with PIMD. All parents gave their informed written consent for their child to take part. The study was conducted in accordance with the Declaration of Helsinki and its experimental protocol had been approved by the Ethics Committee of the University of Geneva.

The control group was composed of 32 TD children (14 girls, 18 boys), aged 12 to 36 months (*M* = 22.53, *SD* = 7.46). They were all French-speaking, coming from families with low to high socioeconomic status (SES). Families’ SES, calculated as a percentage using the formula proposed by the Swiss author Genoud (2011) [54], ranged from 15% (referring to the lower class) to 85% (i.e., the upper class). The average SES was 67% (*SD* = 17.7) corresponding to the middle class. Participants lived in rural areas (< 2000 inhabitants; *n* = 7), villages (2000–5000 inhabitants; *n* = 3), and urban areas (5001 to + 20’000 inhabitants; *n* = 22) in the surroundings of Geneva, Switzerland. Their parents were between 24 and 47 years old (*M* = 34.13, *SD* = 5.60).

This single-case study included nine Caucasian children and adolescents (six girls and three boys, aged 6–17 years) with a diagnosis of PIMD. They all met the criteria set by the internationally accepted definition that the PIMD population consists of individuals with a profound intellectual disability in combination with a severe or profound motor disability [2]. Individual profiles of each of the nine participants are described below. Overall, none of them had functional verbal communication. They also all experienced severe motor limitations in their movement, not walking autonomously, and were thus totally dependent on support persons for all activities of daily life. Each was enrolled in the same Medical-Educational Institute (MEI) of the French Red Cross, located in the department of Haute Savoie (France).

Each participant’s cognitive skill profile was established by a psychologist using the revised version of the “P2CJP questionnaire” [55]: the ECP scale (Evaluation-Cognition-PIMD) [56]. This recent francophone observation-based instrument, was developed by a group of academics and clinical psychologists to assess a wide range of competencies and thus establish valid individual profiles of the following eight cognitive skills explored through 62 items: sensory skills, attention, memory, communication, reasoning, spatial-temporal skills, learning and social-emotional skills. The MEI staff were all familiar with this instrument before the implementation of the present study as they were already using it in their daily practice to define the objectives of each resident and to follow up on individual progress. Individual standardized scores (*t*-scores) on the ECP subscales are presented in Table 1.

**Table 1:** Standardized scores (*t*-scores) on the ECP Scale of the nine PIMD participants.

ECP subscales	Children (< 13 years old)						Adolescents (13 to 18 years old)						
	#1 Zoe		#2 Nina	#3 Suzan		#4 Jane	#5 Betty		#6 Elise	#7 Mike		#8 John	#9 Tim
Sensory	56	51^Pa^^.^	46	36	36^Pa^^.^	41	44	35^Pa^^.^	46	61	50^Pa^^.^	42	50
Attentional	68	74^Pa^^.^	70	46	48^Pa^^.^	66	50	50^Pa^^.^	68	69	65^Pa^^.^	60	63
Memory	60	57^Pa^^.^	60	40	31^Pa^^.^	44	48	54^Pa^^.^	50	70	70^Pa^^.^	66	54
Communicative	57	59^Pa^^.^	52	36	33^Pa^^.^	47	51	50^Pa^^.^	54	65	69^Pa^^.^	60	50
Reasoning	44	42^Pa^^.^	52	37	31^Pa^^.^	47	51	49^Pa^^.^	56	62	56^Pa^^.^	65	54
Spatio-temporal	58	58^Pa^^.^	46	33	32^Pa^^,^	58	52	55^Pa^^.^	44	69	66^Pa^^.^	40	47
Learning	50	47^Pa^^.^	50	36	33^Pa^^.^	53	46	44^Pa^^.^	50	58	53^Pa^^.^	55	52
Social-emotional	60	62^Pa^^.^	62	49	43^Pa^^.^	56	52	54^Pa^^.^	65	67	65^Pa^^.^	64	60

^Pa^. Standardized t-scores computed on parents’ observations. In addition to the psychologist’s assessment, the parents of participants #1 (Zoe), #3 (Suzan), #5 (Betty), and #7 (Mike) also completed the ECP Scale.

**Participant #1: Zoe** was a 6-year-8-month-old girl diagnosed with PIMD due to neonatal microcephaly caused by a mutation in the ASNS gene. This specific genetic anomaly responsible for Zoe’s microcephaly was only identified in 2013 [57]: by 2018, no more than thirty cases had been described in the scientific literature [58]. Zoe presented axial hypotonia and peripheral hypertonia of the lower limbs with equinus feet. Despite this, she was able to crawl freely in her living space and was also capable of sitting up on her own. Zoe had no particular sensory deficits and showed relatively high attentional skills.

**Participant #2: Nina** was an 8-year-old girl diagnosed with PIMD due to a Rett syndrome (RTT) resulting from a mutation in the MECP2 gene. Regarding the RTT cascade of clinical symptoms delineated in a staging system by Engerström (1990) [59], Nina entered the Late Motor Deterioration stage (stage IV) since she ceased walking and became wheelchair-dependent. She presented a severe loss of muscle tone (hypotonia), postural stiffness, and varus feet. Nina also showed stereotyped hand movements, breathing irregularities (hyperventilation during wakefulness, forced expulsion of air and saliva), and a seizure disorder. She had no particular sensory deficits except for a strabismus and slight hyperopia corrected by wearing glasses. Nina showed remarkable eye contact and visual pointing behaviors, and appeared to have high attentional skills.

**Participant #3: Suzan** was an 8-year-11-month-old girl diagnosed with PIMD due to a hypoxic-ischemic encephalopathy (HIE) resulting from a sudden infant death from which she was rescued at the age of 4 months. She presented spastic tetraparesia associated with significant hypotonia. Her secondary deficits included severe epilepsy, right hip dislocation, and sleeping disorders. Suzan tended to be quite floppy, showing minimal reactions to sights or sounds, and exhibiting fluctuating abilities. She had no particular sensory deficits other than the fact that she was often hypothermic. She had relatively low scores on almost all ECP subscales that were consistent between the psychologist’s and her parents’ assessment.

**Participant #4: Jane** was a 9-year-11-month-old girl diagnosed with PIMD due to Nicolaides-Baraitser syndrome caused by a heterozygous missense mutation in the SMARCA2 gene combined with a Chiari type 1 malformation. Despite ankle dorsiflexion, equinovarus feet, and rheumatological disorders, Jane was able to walk (crouching gait) short distances with the assistance of a support person to move freely around a room or to reach a goal. She had no particular sensory deficits and exhibited relatively high attentional skills.

**Participant #5: Betty** was a young, 13-year-old adolescent girl diagnosed with PIMD due to infantile epileptic encephalopathy with hypsarrhythmia identified as idiopathic West syndrome (also known as infantile spasms). Betty’s severe generalized tonic-clonic seizures (consisting of series of sudden involuntary muscle contractions in flexion) first appeared when she was 5 months old, although she had no genetic abnormalities or any brain damage. She presented axial hypotonia (mainly in the extensor muscles of the neck and spine, leading to a global kyphosis of the latter) and peripheral hypertonia (stiffness of the lower limbs). She had no particular sensory deficits but exhibited significant, challenging, self-aggressive behaviors and stereotyped movements of the upper limbs. Betty showed very limited attentional skills resulting in difficulties in maintaining eye contact and decreased responses to sound stimuli.

**Participant #6: Elise** was a 14-year-6-month-old teenage girl diagnosed with PIMD due to early infantile epileptic encephalopathy (EIEE) with suppression-burst, also known as Ohtahara syndrome, caused by a heterozygous missense mutation in the STXBP1 gene. She presented hypertonia, spasticity, dorso-lumbar scoliosis, hip subluxation as well as severe and irreducible foot deformities: equinovalgus left foot and varus right foot. As associated secondary disorders, Elise had epilepsy (treated and stabilized for one year), early onset of puberty (from the age of 8 years), and chronic constipation. She had no particular sensory deficits except for reflexive startle movements to noises (accentuated by fatigue).

**Participant #7: Mike** was a 15-year-old teenage boy diagnosed with PIMD due to neonatal epileptic encephalopathy (NEE) caused by a heterozygous pathogenic variant in the KCNQ2 gene. He presented axial hypotonia and spastic quadriplegia yet he was able to walk a few meters with support (crouch gait), make the transition from sitting to standing, and stand for a while, especially when changing posture (e.g., from his wheelchair to other support devices), during care or dressing. Mike exhibited several stereotyped behaviors but no particular sensory deficits. Consistently, he scored rather high on almost all ECP subscales both through the assessment of the psychologist and his parents. In addition, he had great eye contact and very precise visual pointing behaviors. Combined with his cognitive performance, Mike’s accurate ocular movements enabled him to start learning to use an alternative communication system to express his choices by selecting corresponding pictograms.

**Participant #8: John** was a male adolescent of 16 years and 8 months diagnosed with PIMD due to an Aromatic L-amino acid decarboxylase (AADC) deficiency, consisting in a rare autosomal recessive neurometabolic disorder. John presented severe hypotonia, spasticity and muscle stiffness. He had no particular sensory deficits but exhibited many uncontrolled movements (oculogyric crisis, dystonia, and hypokinesia), and autonomic symptoms such as impaired sweating, nasal congestion, drooling, hypotension, and severe gastroesophageal reflux. John had relatively high cognitive skills, especially in memory (visual and auditory) and in reasoning. Through the regular use of computer-based educational programs over the past two years, John’s understanding of causal relationships has improved significantly. He also had good social-emotional skills. John was very expressive (showing his joy, happiness and interest but also his disagreement and opposition through different reactions), joking and receptive to humor and irony.

**Participant #9: Tim** was a 17-year-old male adolescent diagnosed with PIMD due to Ring chromosome 14 syndrome, characterized by early onset refractory epilepsy, intellectual disability, autism spectrum disorder (whereby Tim was first diagnosed with ASD at the age of 4), and a number of diverse health problems such as dorso-lumbar scoliosis, flexion contractures, early arthritis, recurrent pneumonia and respiratory tract infections. Nevertheless, Tim did not have any additional sensory deficits and showed fairly good attentional and social-emotional skills.

### Apparatus and procedure

Children in the control group completed a single individual eye-tracking session at Geneva’s BabyLab. After having read and approved the consent form and completed a brief socio-demographic questionnaire, the accompanying parent placed his/her child comfortably in front of the screen, either in a suitable seat or on his/ her lap (in which case the parent wore opaque glasses to avoid any interference during the recording of the child’s eye movements). All parents were instructed not to move nor intervene for the duration of the experiment (unless necessary). The eye-tracking session, whose content is detailed in the Stimuli section, could then begin. Once finished, children received a medal as a thank you for participating.

Participants with PIMD each completed between 5 and 8 sessions at best (i.e., with participants who were more available, had less care or activities scheduled in their individual agenda and/or those who were more often in a state of health and alertness suitable to perform a session). Experiments took place in a quiet room of the MEI dedicated to eye-tracking use. The sessions were never scheduled in advance: the experimenter went to the MEI one to two days a week and conducted sessions with the participants as soon as one of them was available and suitable to perform one. A period of three months was necessary for all nine participants to have completed at least five sessions (i.e., the minimum number that had been set beforehand).

The individual eye-tracking sessions performed by the control group and by the PIMD participants were strictly identical. The ocular movements of both populations were measured using the same eye-tracking device: the infrared corneal reflection eye-tracker Tobii X3-120 (Tobii Pro AB, Danderyd, Sweden). Only the size of the presentation screen differed between the two populations. A 22’’ screen (47.5 x 30 cm) was used with the control group and another 23’’ one (50.9 x 28.6 cm) with the PIMD participants. Both displays had a spatial resolution of 1920 x 1080 pixels. Nevertheless, the difference in size between the two screens did not affect the required distance between participants’ eyes and the eye-tracker (magnetized to the lower side of the screen), which had to be 60 to 65 cm (i.e., Tobii’s recommendation for minimizing the impact of head movements on gaze data). Consequently, the gaze angle (α) remained the same with both displays (α ≃ 31.5°), having no impact on the recorded data, thus making them fully comparable. During the experiment, light conditions were kept low to minimize visual distraction.

A session consisted of the following elements: (a) a 30-second opening cartoon (to focus participants’ attention), (b) a 3-point calibration procedure (i.e., completed when 3 points were successfully calibrated and repeated for the missing ones) in which an expanding-contracting circle appeared in every position of a 2 x 3 grid of white dots on a black background (a satisfactory calibration was achieved with less than 2° of deviation on the x and y axes), (c) the experimental phase, and (d) a brief final animation (a puppet thanking the participant and saying goodbye).

The experimental phase consisted of the successive presentation of various randomly ordered visual stimuli, divided into six ‘blocks’ of experimental content (each underpinning one of the six social-emotional abilities assessed). Between each block, a dynamic fixation cross paired with a stimulating sound was presented in the screen’s center until the participant looked at it (i.e., the 400 x 400 pixels AOI drawn around it had to be gazed at for at least 300 ms to trigger the next block) in order to prevent a side bias from compromising the reliability of the recorded data. This fixation cross was also presented between each of the trials composing the different blocks, but this time for a fixed duration (1500 ms) that no longer depended on participants’ gaze. Consequently, we checked post hoc whether they had looked at it between each trial or not: in this case, the concerned trial(s) were removed from the analyses.

The total duration of a session (including the opening cartoon, the calibration, the experimental phase, and the brief final thank-you message) was about 6 minutes. It could be longer if the calibration had to be repeated and/or if the participant did not look directly at the screen’s center between blocks (thus delaying their activation), but it never exceeded 10 minutes.

### Stimuli

To measure the social-emotional abilities of interest, we integrated six ‘blocks’ of experimental content into our paradigm. This content consisted of visual stimuli specifically chosen for its variability. The variability related to the visual characteristics of the stimuli (high/low contrast, black and white/colored, dynamic/static, sparse/complex, or with many/few details), but also the screen area they occupied (covering the whole screen or only a part of it), the level of abstraction of their content (abstract forms vs. concrete situations, objects, or individuals), and their presentation modalities (few trials with long presentation times vs. many trials with shorter presentation times).

Since we decided to use a visual preference procedure involving the simultaneous presentation of two side-by-side stimuli, each task had a minimum of two trials in order to counterbalance the pairs of stimuli laterally.

**The "Point-Light (PL)-Task"** of our paradigm used the stimuli created by Bidet-Ildei, Kitromilides, Orliaguet, Pavlova, and Gentaz (2014) to measure our participants’ ability to discriminate biological motion from non-biological motion [38]. It included two 20-second trials (for a total duration of approx. 42 seconds). The stimuli pairs thus presented on each trial consisted of moving white dots depicting a human point-light walker (biological motion) beside another point-light set of randomly moving dots (non-biological motion). The AOI drawn around each stimulus was 960 x 1080 pixels (covering the entire screen). Based on a previous eye-tracking study [60], participants had to look at the display for at least 500 ms for a trial to be validated and its data processed.

**The "Social Orienting (SO)-Task"** of our paradigm used the stimuli created by Franchini et al. (2016) [32], to measure the ability to discriminate socially salient visual scenes from non-social scenes in our participants. It included eight 5-second trials (for a total duration of approx. 48 seconds considering inter-trial delays). The stimuli pairs thus presented consisted of dynamic social images (sequences showing a young boy or girl moving and dancing solo) beside dynamic geometric images (moving geometrical shapes similar to classic abstract screen savers). The AOI drawn on each stimulus was 815 x 1080 pixels (corresponding to half of the screen minus the black stripe area on the edges of the original stimuli). As in the previous task, participants had to look at the display for at least 500 ms for a trial to be validated and its data processed.

**The male and female emotional (happy and angry) faces** used to test our participants’ visual scanning of facial features and emotion discrimination came from the “The Karolinska Directed Emotional Faces—KDEF”, references F22 and M17 [61]. This selection was based on the validation article of this database [62]: these were the only models in the 20 best joy and anger pictures established by the authors to have teeth visible, as teeth have been shown to be a particularly salient facial feature influencing emotion discrimination [63]. To increase ecological validity [64] the stimuli were presented in color on a medium gray background (RGB 100, 100, 100). The hairline, another facial feature known to affect the visual exploration of faces [65], was cropped using GIMP software.

Since we aimed to assess two distinct abilities from these two pairs of faces, we allocated one experimental ‘block’ (consisting of two 20-second trials) to each. Different AOIs were drawn as the two abilities were actually measured by comparing the time spent looking at different locations: (a) time on the eye area (composed of two AOIs of 160 x 160 pixels each, spaced 140 pixels apart) vs. the mouth area (consisting of an AOI of 320 x 160 pixels, equivalent to the size of the eye region) for visual scanning of facial features, and (b) time on happy face vs. angry face (i.e., on each of the two 600 x 790 pixels AOIs, symmetrically located at a distance of 180 pixels from the screen center) for emotion discrimination.

Trial validation also depended on looking times at the different AOIs: for visual scanning, participants had to have looked at the three AOIs (i.e., mouth, left and right eye areas) for at least 200 ms, whereas for emotion discrimination they had to have looked at the two AOIs delimiting both emotional faces for at least 300 ms.

**The "RJA-Task"** of our paradigm used the stimuli created by Franchini et al. (2017) [49] to test participants’ ability to follow the direction of others’ gaze to share a common point of reference. Only the videos of the ‘intense’ condition that had previously generated significantly higher looking times were integrated into our experimental paradigm: they consisted of four 15-second videos in which an actress was standing behind a table with two identical objects (of different natures, shapes, and colors in each video). In this initial position, the actress was looking directly at the camera. After 4 seconds, the objects started to move, she then suddenly focused her attention on one of them with an expression of intense surprise that lasted 10 seconds. The AOI was drawn on each of the two 450 x 350 pixels objects (the one that was looked at and the one that was not). Both AOIs were symmetrically located at a distance of 225 pixels from the center of the screen length. An additional area (of identical size) was also drawn around the actress’ face in order to control for the quality of the visual exploration: participants had to look at it for at least 300 ms for a trial to be validated and its data processed.

**The "SME-Task"** of our paradigm was an adaptation of the “Climbing the hill” paradigm [51], in which a ‘climber’ puppet (a red, circular wooden character with large plastic ‘googly’ eyes) tried but failed to climb a steep hill, and was randomly either bumped up the hill by the ‘helper’ (prosocial behavior) or bumped down the hill by the ‘hinderer’ (antisocial behavior). Each of these “climbing scenes” lasted 15 seconds. This first part of the task was followed by a final visual preference scene (consisting of two 15-second trials), in which both the helper and the hinderer puppets (a blue square and a yellow triangle with googly eyes) were simultaneously presented side by side on the entire screen surface (delineated by two 960 x 1180 pixels AOIs). Consequently, our SME-Task differed from the others by its structure (the visual preference scene was only a part of the task, occurring only after the presentation of the first two climbing scenes), but also by the fact that the visual preference measurement depended on the quality of the visual exploration of the previous climbing scenes: the participants had to have looked at each of them for at least 5 seconds and the difference in their respective looking times had to be no more than 10 seconds for a trial to be valid. Since these inclusion criteria might eliminate a number of trials, participants’ socio-moral evaluation ability was also measured by comparing the time spent gazing at each climbing scene to determine whether one of the two observed behaviors (pro- vs. anti-social) attracted more visual attention.

### Data analysis

Raw data were extracted using Tobii Pro Lab (Version 1.162) computer software. We were interested in the total looking times (LT) at all the AOIs drawn on every stimulus, computed by this software as the total visit duration. Visits are defined as the portion of gaze data between the start of the first fixation on the AOI until the end of the last fixation on the AOI, before an exit saccade. Fixations can be defined as the periods of time where the eyes are relatively still, holding the central foveal vision in place so that the visual system can take in detailed information about what is being looked at, whereas saccades refer to rapid, ballistic movements of the eyes that abruptly change the point of fixation [27]. These raw times (in seconds) were relevant primarily for exploring and comparing the general characteristics of individual oculomotor behaviors in response to different visual stimuli.

Nevertheless, statistical analyses were not performed on these raw data but on proportions of looking times (expressed as LT percentages), which were computed on different ratios depending on what was being tested: (1) for feasibility, proportions of LTs on the different blocks/tasks of the paradigm relative to their respective durations; (2) for visual preferences, proportions of LTs on one of the paired stimuli (or on a specific AOI) relative to the LTs on both stimuli (or on all the specific AOIs compared).

First, regarding the feasibility of the paradigm, we started by running preliminary analyses consisting of descriptive statistics (means, standard deviations, ranges) of total LT percentages of both the PIMD individuals at each session performed, and the control group. We then focused on the individual data of the PIMD participants and performed a within-subject analysis of variance (ANOVA) of the mean LT percentages to test the effect of the blocks’ order of presentation (regardless of their specific content), the effect of fatigue between the first two and the last two blocks presented (through complementary post-hoc contrast analyses), and the effect of the blocks’ content (i.e., task-specific visual stimuli). Additionally, before proceeding with the analysis of visual preferences, the symmetry (left/right side of the screen and upper/lower part of it) of the participants’ visual exploration was examined using the data from the opening cartoon and the final animation. We extracted the number of fixations on each of these four screen areas, calculated the ratios, and verified that there was no significant asymmetry in individual exploration of the screen.

Second, regarding the visual preferences, we started by excluding all individual trials that failed to meet the task-specific inclusion criteria from the analyses (c.f. Stimuli Section), as well as those whose priming fixation cross presented in the center of the screen had not been gazed at, before proceeding to skill-by-skill and case-by-case data processing. Then, the LT percentages on each of the paired stimuli (or on a specific AOI) of both the PIMD individuals and the control group were compared either through paired-sample *t*-tests (when the distribution of our dependent variables was normal) or non-parametric Wilcoxon matched-pair tests (when the distribution was not normal). Whenever the mean LT percentage was significantly higher on one of the two stimuli/AOIs compared, we concluded that there was a visual preference for it. Normality was tested using the Shapiro-Wilk W-test.

All statistical analyses were conducted using TIBCO Statistica (version 13.2). A *p*-value ≤ 0.05 was considered significant.

## Results

### Feasibility of the eye-tracking-based experimental paradigm

Considering the general characteristics of the eye-tracking data of the two populations studied, we first noted that PIMD participants spent overall less time looking at the screen compared to the control group (Mann-Whitney *U* = 342, *p* < .001), thereby justifying the fact that we calculated percentages of looking times (at the screen and then at specific AOIs) and performed the statistical tests on these proportions rather than on the raw times.

#### Preliminary analyses: Descriptive statistics of LT percentages

Table 2 presents the percentages of time each of the nine PIMD participants spent looking at the screen in the different sessions they individually performed (over the session duration), as well as the total mean LT percentage for each of them and for the control group. We observed that seven of the nine participants spent more than half the time looking at the screen (LT percentages > 50%) by the very first session (S1): only participants #5 (Betty) and #6 (Elise) spent more time looking away from the screen during its completion (respective LT percentages of 24.06 and 15.09). While the gaze rate of participant #6 (Elise) was indeed particularly low at S1, it increased considerably at S2 (48.88%), reached the 50% threshold at S3 (51.96%), and decreased again in the last two sessions (30.39 and 20.80%). This variability was not found in the session percentages of participant #5 (Betty). With a mean LT percentage of 29.22 (*SD* = 4.76), Betty appeared to be the participant who spent, on average, the least amount of time looking at the screen during the five sessions she performed. Additionally, this extremely low gaze rate tended to be especially consistent across sessions: no other participant obtained such a small standard deviation. Although relatively little variability was also observed in the session LT percentages of participant #1 (Zoe), which ranged from 45.05 (at S7) to 67.52 (at S1) (*M* = 54.15%, *SD* = 8.44).

**Table 2:** Preliminary analyses: Descriptive statistics of looking times (LT) of the nine PIMD participants and the control group (session percentages, means, standard deviations, and ranges).

Participant	LT percentage^1^ at each session performed								Mean *(SD)*		Range
	S1	S2	S3	S4	S5	S6	S7	S8			
# 1 Zoe	67.52	50.23	54.09	49.69	65.45	45.66	45.05	55.49	54.15	*(8*.*44)*	45.05–67.52
# 2 Nina	73.29	51.24	69.07	85.77	70.74	75.35	90.20	46.61	70.28	*(15*.*10)*	46.61–90.20
# 3 Suzan	55.14	24.61	67.60	17.51	41.78				41.33	*(20*.*79)*	17.51–67.60
# 4 Jane	54.66	83.15	61.24	45.41	18.96				52.69	*(23*.*43)*	18.96–83.15
# 5 Betty	24.06	27.68	35.28	26.02	33.04				29.22	*(4*.*76)*	24.06–35.28
# 6 Elise	15.09	48.88	51.96	30.39	20.80				33.41	*(16*.*49)*	15.09–51.96
# 7 Mike	78.37	29.56	89.08	86.12	59.27	76.46	69.18	81.82	71.23	*(19*.*32)*	29.56–89.08
# 8 John	52.30	22.30	57.11	37.04	69.20				47.59	*(18*.*24)*	22.30–69.20
# 9 Tim	94.03	46.14	64.49	25.78	60.90				58.27	*(25*.*14)*	25.78–94.03
Control Group (*n* = 32)									75.82	*(17*.*28)*	27.22–97.53

^***1***^. Calculated as the proportion of time spent looking at the presentation screen over the session duration.

In contrast, the gaze rates of participants #4 (Jane), #8 (John), and #9 (Tim), whose mean LT percentages were close to Zoe’s (52.69, 47.59 and 58.27, respectively) were much less consistent across the different sessions they individually performed (with respective standard deviations of 23.43, 18.24, and 25.14). Participant #9 (Tim) had the widest range of LT percentages: from only 25.78 in the 4th session, up to 94.03 in the 1st one, which is the highest gaze rate measured in the PIMD individuals.

Nevertheless, some variability was also found in the control group, whose LT percentages ranged from 27.22 to 97.53. With a total mean LT percentage of 75.82 (*SD* = 17.28), it seemed that the variability observed within the control group was very close to the PIMD participants’ intra-individual variability (with a tendency for the latter to be slightly lower). Finally, it should be noted that two PIMD participants have a mean LT percentage very similar to that of the control group: these are #2 (Nina) (*M* = 70.28, *SD* = 15.10), and #7 (Mike) (*M* = 71.23, *SD* = 19.32).

#### Effects of block presentation (order and fatigue) and task content

For the different effects tested, focusing first on those related to the presentation of the blocks (see Table 3), we observed that the repeated measures ANOVA performed to test the order effect was significant only in one PIMD individual: participant #1 (Zoe), *F*(5,35) = 3.442, *p* = .012. A post-hoc analysis (Tukey HSD tests) revealed that, on average, Zoe tended to look significantly longer at the 4th block presented to her (highest mean LT percentage: 63.94, *SD* = 12.83) than the 5th one (lowest mean LT percentage: 44.48, *SD* = 9.2), *p* = .018.

**Table 3:** Mean percentage (and standard deviation) of looking times (LT) at each of the 6 blocks composing a session of both the nine PIMD participants and the control group, and statistical analyses for the two presentation effects (order and fatigue).

Participant	*N*	Mean LT percentage^1^ *(SD)*						Presentation effects	
		Block 1	Block 2	Block 3	Block 4	Block 5	Block 6	Order^2^	Fatigue^3^
# 1 Zoe	8	61.83 *(8*.*74)*	49.90 *(14*.*41)*	52.08 *(13*.*56)*	63.94 *(12*.*83)*	44.48 *(9*.*2)*	57.31 *(17*.*7)*	*F*(5,35) = 3.442*	*t*(7) = 0.940
# 2 Nina	8	73.80 *(13*.*41)*	72.69 *(18*.*2)*	68.97 *(15*.*67)*	72.56 *(18*.*19)*	70.13 *(28*.*57)*	67.60 *(28*.*58)*	*F*(5,35) = 0.192	*t*(7) = 0.614
# 3 Suzan	5	51.64 *(31*.*67)*	35.68 *(37*.*48)*	52.92 *(26*.*47)*	35.29 *(15*.*66)*	35.29 *(20*.*92)*	35.93 *(22*.*1)*	*F*(5,20) = 1.288	*t*(4) = 0.870
# 4 Jane	5	54.25 *(32*.*83)*	49.29 *(26*.*32)*	59.55 *(22*.*66)*	49.03 *(37*.*88)*	41.94 *(35*.*02)*	55.04 *(26*.*49)*	*F*(5,20) = 0.438	*t*(4) = 0.300
# 5 Betty	5	20.03 *(12*.*62)*	13.20 *(9*.*09)*	27.91 *(11*.*16)*	37.90 *(22*.*12)*	46.08 *(41*.*01)*	47.71 *(33*.*46)*	*F*(5,20) = 1.455	*t*(4) = -3.838*
# 6 Elise	5	45.30 *(33*.*26)*	26.96 *(13*.*77)*	19.82 *(10*.*53)*	28.72 *(26*.*5)*	44.97 *(34*.*25)*	36.22 *(28*.*81)*	*F*(5,20) = 1.202	*t*(4) = -0.669
# 7 Mike	8	69.62 *(29*.*64)*	74.99 *(19*.*71)*	67.28 *(18*.*32)*	78.99 *(25*.*24)*	76.298 *(30*.*05)*	57.21 *(21*.*78)*	*F*(5,35) = 1.466	*t*(7) = 1.251
# 8 John	5	33.66 *(23*.*14)*	46.37 *(25*.*62)*	46.08 *(18*.*69)*	57.67 *(28*.*65)*	52.46 *(22*.*12)*	45.55 *(14*.*23)*	*F*(5,20) = 1.356	*t*(4) = -2.499
# 9 Tim	5	69.71 *(16*.*37)*	51.65 *(32*.*33)*	60.83 *(26*.*26)*	55.87 *(32*.*78)*	60.03 *(30*.*12)*	49.88 *(29*.*96)*	*F*(5,20) = 1.213	*t*(4) = 0.750
CG (*n* = 32)		72.98 *(22*.*03)*	78.44 *(23*.*66)*	76.59 *(24*.*30)*	72.43 *(27*.*07)*	74.80 *(22*.*72)*	77.96 *(23*.*70)*	*F*(5,155) = 0.601	*t*(31) = -0.237

^***1***^. Calculated as the time spent looking at the screen during the presentation of each block at the different sessions performed divided by the blocks’ duration.

^***2***^. Main effect of the order of presentation of the six blocks tested using within-subject repeated measure ANOVAs.

^***3***^. Fatigue effect tested using contrast analyses comparing the individual mean LT percentages on the first two blocks presented vs. the last two.

* *p* < .05.

A contrast analysis testing for a possible fatigue effect during the completion of the sessions (comparing the individual LT percentages on the first two blocks presented with those on the last two) was also significant in only one PIMD individual: participant #4 (Betty). However, this was not a fatigue effect since Betty tended to look significantly longer at the last two blocks (*M* = 46.89%, *SD* = 11.95) than at the first two (*M* = 16.61%, *SD* = 6.79), *t*(4) = -3.838, *p* = .018.

Consistent with the results for most PIMD participants, neither the order effect nor the fatigue effect was significant in the control group.

Regarding the effect of the content, the ANOVA conducted to test whether gaze rates depended on the visual stimuli specific to each block was significant for two PIMD participants as well as for the control group (see Table 4). Participant #4 (Jane) spent less time on average looking at the stimuli from the PL-Task (*M* = 32.33%, *SD* = 22.74) than those from the SO-Task (*M* = 63.29%, *SD* = 18.69), *F*(5,20) = 3.379, *p* = .023. Participant #7 (Mike) spent, on average, less time looking at the PL-Task (*M* = 52.47%, *SD* = 16.28) than at both the SO-Task (*M* = 78.46%, *SD* = 31.94) and the RJA-Task (*M* = 82.24%, *SD* = 22.95), *F*(5,35) = 3.104, *p* = .02. Finally, TD children spent significantly more time looking at the stimuli from the SO-Task (*M* = 80.87%, *SD* = 17.0) than at the pair of happy and angry female faces (*M* = 68.16%, *SD* = 25.03), *F*(5,155) = 2.958, *p* = .014.

**Table 4:** Mean percentage (and standard deviation) of looking times (LT) on each of the 6 tasks composing a session of both the nine PIMD participants and the control group, and statistical analyses testing the main effect of the content, using within-subject repeated measures ANOVAs.

Participant	*N*	Mean LT percentage^1^ *(SD)*						Content effect
		PL-Task	SO-Task	Female faces	Male faces	RJA-Task	SME-Task	
# 1 Zoe	8	56.48 *(17*.*50)*	57.83 *(15*.*69)*	45.93 *(10*.*80)*	55.44 *(14*.*76)*	49.73 *(12*.*02)*	64.13 *(9*.*10)*	*F*(5,35) = 2.241
# 2 Nina	8	65.85 *(22*.*97)*	69.12 *(17*.*77)*	77.43 *(15*.*47)*	71.55 *(20*.*58)*	64.94 *(15*.*21)*	76.85 *(29*.*21)*	*F*(5,35) = 1.036
# 3 Suzan	5	46.97 *(26*.*34)*	30.03 *(17*.*24)*	45.11 *(9*.*97)*	52.50 *(32*.*94)*	38.94 *(26*.*60)*	33.21 *(37*.*45)*	*F*(5,20) = 1.267
# 4 Jane	5	32.33 *(22*.*74)*	63.29 *(18*.*69)*	37.58 *(26*.*29)*	59.81 *(31*.*64)*	60.71 *(31*.*48)*	55.36 *(35*.*29)*	*F*(5,20) = 3.379*
# 5 Betty	5	23.84 *(13*.*81)*	24.26 *(9*.*80)*	31.07 *(26*.*0)*	39.66 *(20*.*48)*	15.04 *(13*.*14)*	58.94 *(42*.*94)*	*F*(5,20) = 1.90
# 6 Elise	5	45.35 *(38*.*27)*	36.90 *(21*.*96)*	20.96 *(9*.*31)*	20.11 *(14*.*37)*	35.98 *(21*.*61)*	42.68 *(36*.*68)*	*F*(5,20) = 1.347
# 7 Mike	8	52.47 *(16*.*28)*	78.46 *(31*.*94)*	67.40 *(24*.*67)*	69.03 *(23*.*89)*	82.24 *(22*.*95)*	74.76 *(18*.*67)*	*F*(5,35) = 3.104*
# 8 John	5	49.62 *(20*.*24)*	47.89 *(19*.*70)*	44.37 *(24*.*13)*	36.76 *(24*.*62)*	57.79 *(28*.*46)*	45.37 *(19*.*11)*	*F*(5,20) = 0.913
# 9 Tim	5	55.44 *(24*.*51)*	57.0 *(23*.*73)*	62.36 *(30*.*98)*	46.82 *(30*.*46)*	65.81 *(31*.*39)*	60.54 *(29*.*94)*	*F*(5,20) = 0.975
CG (*n* = 32)		69.55 *(27*.*51)*	80.87 *(17*.*0)*	68.16 *(25*.*03)*	77.57 *(17*.*68)*	80.31 *(26*.*41)*	76.75 *(25*.*40)*	*F*(5,155) = 2.958*

^***1***^. Calculated as the time spent looking at the screen during each task’s completion at the different sessions performed divided by the tasks’ duration.

* *p* < .05.

In addition, it is interesting to note that the individual gaze rates presented above are related to scores on the attentional skills subscale of the ECT questionnaire (measured through items such as "Voluntarily directs attention ‘to *a person*’ or ‘to *an object of interest*‴ and "Maintains sustained attention"). Pearson correlations then showed that attentional skills were significantly associated with the mean LT percentage in each session (*r* = .454) and with the mean LT percentage in the following tasks (*p* < .05): PL-Task (*r* = .328), SO-Task (*r* = .539), emotional faces (*r* = .262), RJA-Task (*r* = .439) and SME-Task (*r* = .312).

#### Visual exploration symmetry

In most eye-tracking studies, participants are assumed to be able to explore the entire screen surface based on their calibration output, but as we opted for a low-demand procedure (only 3 successful points) in order to maintain participants’ attention, the analysis of the distribution of fixations on the screen during both the opening cartoon and the final animation completes and strengthens this undemanding procedure by providing additional information regarding participants’ visual exploration symmetry.

Overall, the fixations of both the control group and the PIMD participants were symmetrically distributed over the entire screen surface, resulting in small differences between the percentages of fixations on the left and right side of the screen, as well as between on the upper and lower part of it. Differences greater than 10% were observed in only two individuals. Participant #7 (Mike) showed the highest difference between his visual exploration of the left vs. right side of the screen (respective mean fixation percentages: 55.5 vs. 44.5%, *SD* = 3.23). Regarding the visual exploration of the upper versus lower part of the screen, the greatest difference in fixation percentages was shown by participant #8 (John) (*M* = 56.2 vs. 43.8% respectively, *SD* = 2.94). This result appears to be consistent with calibration outputs indicating that the completion of this 3-point procedure was systematically problematic with participant #8 (John) (e.g., recurrent insufficient data collected for each calibration point, often remaining poorly accurate even once validated).

### Visual preference in the six social-emotional abilities

#### Biological vs. non-biological motion

The control group showed no visual preference for biological or non-biological motion. Although TD children tended to look slightly longer at biological motion than at non-biological (51.75 vs. 48.25%, *SD* = 23.62), this difference was not significant (*t*(58) = 0.568, *p* = .572). Similarly, the difference between the LT percentages for the two types of stimuli of the PL-Task was not significant for any of the PIMD individuals. However, the same tendency to look longer at biological motion was found in seven of them. Only participants #3 (Suzan) and #8 (John) showed the opposite pattern, resulting in higher LT percentages on non-biological motion. Results are detailed in Table 5.

**Table 5:** Mean (and *SD*) looking times (raw and percentage) at each of the two stimuli of the PL-Task (i.e., biological vs. non biological motion), consisting of two 20-second trials, of the control group and the nine PIMD participants.

	*n valid trials*	Raw looking times (LT) in seconds				LT in proportion (percentage)				Difference in LT percentages	
		Biological motion		Non-biological motion		Biological motion		Non-biological motion			
		*M*	*(SD)*	*M*	*(SD)*	*M*	*(SD)*	*M*	*(SD)*	*t*(n-1)	*p*
Control group (*n* = 30^1^)	59	8.305	*(4*.*653)*	7.021	*(3*.*737)*	51.75	*(23*.*62)*	48.25	*(23*.*62)*	0.568	.572
Participant #											
# 1 Zoe	16	5.857	*(3*.*631)*	5.199	*(3*.*423)*	53.01	*(24*.*26)*	46.99	*(24*.*26)*	0.497	.626
# 2 Nina	16	6.761	*(4*.*441)*	6.812	*(5*.*139)*	51.74	*(27*.*48)*	48.26	*(27*.*48)*	0.253	.804
# 3 Suzan	10	2.519	*(2*.*163)*	6.364	*(5*.*657)*	35.68	*(35*.*03)*	64.32	*(35*.*03)*	-1.293	.228
# 4 Jane	6	5.832	*(4*.*193)*	2.655	*(2*.*886)*	60.71	*(37*.*79)*	39.29	*(37*.*79)*	0.694	.519
# 5 Betty	8	3.725	*(3*.*850)*	1.659	*(1*.*535)*	60.55	*(34*.*82)*	39.45	*(34*.*82)*	0.857	.420
# 6 Elise	10	5.070	*(6*.*318)*	4.254	*(6*.*226)*	50.59	*(43*.*24)*	49.41	*(43*.*24)*	0.043	.967
# 7 Mike	16	4.715	*(2*.*747)*	5.516	*(4*.*431)*	52.71	*(27*.*16)*	47.29	*(27*.*16)*	0.399	.695
# 8 John	10	4.781	*(4*.*497)*	4.725	*(4*.*598)*	48.15	*(38*.*07)*	51.85	*(38*.*07)*	-0.153	.881
# 9 Tim	9	5.696	*(3*.*586)*	6.174	*(6*.*082)*	54.85	*(29*.*24)*	45.15	*(29*.*24)*	0.498	.632

^***1***^. Data from two TD children were excluded from the analyses; the remaining sample consisted of 30 TD children (12 girls, 18 boys) aged 12 to 36 months (*M* = 23.03, *SD* = 7.42).

#### Socially salient vs. non-social scenes

The control group showed a visual preference for socially salient stimuli (*t*(247) = 15.891, *p* < .001). Among the PIMD individuals, this same preference was found in two participants: #1 (Zoe) looked longer at social than at non-social scenes (60.74 vs. 39.26%, *SD* = 25.45; *t*(63) = 3.378, *p* = .001), as did #8 (John), with respective LT percentages of 70.85 and 29.15 (*SD* = 28.02), *t*(37) = 4.587, *p* < .001. Despite non-significant differences, four PIMD individuals exhibited the same tendency as the TD children to look slightly longer at social stimuli: participants #2 (Nina), #4 (Jane), #6 (Elise), and #7 (Mike). Finally, participants #3 (Suzan), #5 (Betty), and #9 (Tim) showed the opposite pattern, resulting in higher LT percentages on non-social scenes. Results are detailed in Table 6.

**Table 6:** Mean (and *SD*) looking times (raw and percentage) at each of the two stimuli of the SO-Task (i.e., social vs. non social scenes), consisting of eight 5-second trials, of the control group and the nine PIMD participants.

	*n valid trials*	Raw looking times (LT) in seconds				LT in proportion (percentage)				Difference in LT	
		Social scenes		Non-social scenes		Social scenes		Non-social scenes		percentages	
		*M*	*(SD)*	*M*	*(SD)*	*M*	*(SD)*	*M*	*(SD)*	*t*(n-1)	*p*
Control group (*n* = 32)	248	3.589	*(1*.*431)*	1.254	*(1*.*239)*	74.27	*(24*.*05)*	25.73	*(24*.*05)*	15.891	< .001
Participant #											
# 1 Zoe	64	1.907	*(1*.*136)*	1.327	*(1*.*054)*	60.74	*(25*.*45)*	39.26	*(25*.*45)*	3.378	.001
# 2 Nina	56	1.976	*(1*.*659)*	1.925	*(1*.*653)*	51.97	*(33*.*88)*	48.03	*(33*.*88)*	0.436	.665
# 3 Suzan	25	0.824	*(0*.*909)*	1.294	*(1*.*480)*	48.25	*(40*.*72)*	51.75	*(40*.*72)*	-0.215	.831
# 4 Jane	39	1.674	*(1*.*685)*	1.930	*(1*.*983)*	53.35	*(37*.*97)*	46.65	*(37*.*97)*	0.551	.585
# 5 Betty	22	1.302	*(1*.*591)*	1.137	*(1*.*164)*	49.85	*(37*.*89)*˟	50.15	*(37*.*89)*˟	-0.019	.985
# 6 Elise	29	1.483	*(1*.*635)*	1.248	*(1*.*250)*	50.01	*(39*.*40)*	49.99	*(39*.*40)*	0.002	.998
# 7 Mike	60	2.301	*(1*.*725)*	2.495	*(1*.*807)*	50.12	*(32*.*80)*	49.88	*(32*.*80)*	0.028	.978
# 8 John	38	1.845	*(1*.*514)*	0.645	*(0*.*823)*	70.85	*(28*.*02)*	29.15	*(28*.*02)*	4.587	< .001
# 9 Tim	35	1.504	*(1*.*468)*	1.759	*(1*.*657)*	47.53	*(37*.*12)*	52.47	*(37*.*12)*	-0.394	.696

^˟^ Since the distribution of these variables was not normal (Shapiro-Wilk W-test = 0.873, *p* = .009), a non-parametric Wilcoxon matched-pair test was performed to compare them, which also turned out to be non-significant (Z = 0.097, *p* = .922).

#### Facial area of the eye vs. the mouth

The control group showed a significant difference in visual scanning of facial features, with a preference for the eye area over the mouth (71.19 vs. 28.81%, *SD* = 25.71; *t*(122) = 9.142, *p* < .001) of male and female emotional (happy and angry) faces (regardless of emotion and gender). In the PIMD participants, this visual preference was also found in four individuals: on average, participants #2 (Nina), #5 (Betty), #7 (Mike), and #9 (Tim) all looked significantly longer at the eye area (63.91, 80.3, 61.34, and 70.54%, respectively) than at the mouth (36.09, 19.7, 38.66, and 29.46%, respectively). Furthermore, among the five participants where the difference was not significant, none showed the opposite pattern: all PIMD individuals tended to look longer at the eyes than the at the mouth area. Results are detailed in Table 7.

**Table 7:** Mean (and *SD*) looking times (raw and percentage) at each of the two facial AOIs (i.e., eye vs. mouth areas) of the control group and the nine PIMD participants recorded during the repeated presentation (four 20-second trials) of pairs of male and female happy and angry faces.

	*n valid trials*	Raw looking times (LT) in seconds					LT in proportion (percentage)				Difference in LT percentages	
		Eye area		Mouth area			Eye area		Mouth area			
		*M*	*(SD)*	*M*	*(SD)*		*M*	*(SD)*	*M*	*(SD)*	*t*(n-1)	*p*
Control group (*n* = 32)	123	4.573	*(3*.*352)*	1.969	*(2*.*400)*		71.19	*(25*.*71)*	28.81	*(25*.*71)*	9.142	< .001
Participant #												
# 1 Zoe	31	0.790	*(0*.*637)*	0.805		*(0*.*953)*	55.58	*(28*.*63)*	44.42	*(28*.*63)*	1.086	.286
# 2 Nina	30	2.012	*(1*.*482)*	1.371		*(1*.*358)*	63.91	*(24*.*16)*	36.09	*(24*.*16)*	3.153	.004
# 3 Suzan	12	0.446	*(0*.*480)*	0.314		*(0*.*347)*	66.94	*(33*.*68)*	33.06	*(33*.*68)*	1.742	.109
# 4 Jane	17	1.107	*(1*.*426)*	0.761		*(0*.*996)*	60.08	*(32*.*99)*	39.92	*(32*.*99)*	1.260	.226
# 5 Betty	14	1.321	*(1*.*490)*	0.251		*(0*.*447)*	80.30	*(19*.*74)*	19.70	*(19*.*74)*	5.745	< .001
# 6 Elise	16	0.247	*(0*.*245)*	0.187		*(0*.*220)*	58.37	*(31*.*65)*	41.63	*(31*.*65)*	1.058	.307
# 7 Mike	31	2.062	*(1*.*704)*	1.424		*(1*.*348)*	61.34	*(23*.*06)*	38.66	*(23*.*06)*	2.738	.010
# 8 John	15	0.647	*(0*.*698)*	0.514		*(0*.*661)*	56.49	*(35*.*65)*	43.51	*(35*.*65)*	0.705	.492
# 9 Tim	13	1.691	*(2*.*121)*	0.449		*(0*.*400)*	70.54	*(29*.*83)*	29.46	*(29*.*83)*	2.483	.029

#### Happy vs. angry faces

The control group showed a significant difference in emotion discrimination, with a visual preference for angry faces over happy faces (56.77 vs. 43.23%, *SD* = 16.57), *t*(124) = 4.57, *p* < .001. No significant difference was found in the LT percentages on any pair of emotional faces in PIMD individuals. However, the majority (five participants) showed a similar tendency to spend more time looking at angry faces, while only four of them showed the opposite pattern. Results are detailed in Table 8.

**Table 8:** Mean (and *SD*) looking times (raw and percentage) at each of the two emotional expressions (i.e., angry and happy faces) of the control group and the nine PIMD participants recorded during the repeated presentation (four 20-second trials) of pairs of male and female happy and angry faces.

	*n valid trials*	Raw looking times (LT) in seconds				LT in proportion (percentage)				Difference in LT percentages	
		Angry face		Happy face		Angry face		Happy face			
		*M*	*(SD)*	*M*	*(SD)*	*M*	*(SD)*	*M*	*(SD)*	*t*(n-1)	*p*
Control group (*n* = 32)	125	6.702	*(3*.*554)*	5.021	*(2*.*828)*	56.77	*(16*.*57)*	43.23	*(16*.*57)*	4.570	< .001
Participant #											
# 1 Zoe	32	2.750	*(1*.*817)*	2.959	*(2*.*311)*	49.29	*(28*.*65)*	50.71	*(28*.*65)*	-0.139	.890
# 2 Nina	32	5.934	*(3*.*843)*	3.930	*(2*.*747)*	57.52	*(25*.*67)*	42.48	*(25*.*67)*	1.657	.108
# 3 Suzan	15	1.297	*(1*.*280)*	2.232	*(1*.*920)*	43.19	*(38*.*46)*	56.81	*(38*.*46)*	-0.686	.504
# 4 Jane	17	2.705	*(2*.*404)*	2.768	*(2*.*269)*	46.81	*(34*.*19)*	53.19	*(34*.*19)*	-0.385	.705
# 5 Betty	16	1.640	*(1*.*907)*	1.927	*(3*.*637)*	52.25	*(42*.*18)*	47.75	*(42*.*18)*	0.213	.834
# 6 Elise	17	1.008	*(1*.*042)*	1.245	*(1*.*597)*	52.60	*(40*.*19)*	47.40	*(40*.*19)*	0.267	.793
# 7 Mike	32	4.154	*(3*.*163)*	5.409	*(3*.*249)*	43.59	*(21*.*43)*	56.41	*(21*.*43)*	-1.693	.101
# 8 John	17	2.534	*(3*.*036)*	2.406	*(3*.*402)*	52.31	*(41*.*66)*	47.69	*(41*.*66)*	0.229	.822
# 9 Tim	14	4.069	*(3*.*869)*	2.538	*(2*.*148)*	54.18	*(34*.*27)*	45.82	*(34*.*27)*	0.457	.655

#### Objects of joint attention vs. non-looked-at objects

The control group showed a significant difference in the amount of time spent looking at the objects that the actress was—or was not—looking at in the RJA-Task, with a visual preference for the looked-at objects (i.e., those reflecting correct RJA behaviors) over the non-looked-at ones (57.98 vs. 42.02%, *SD* = 22.96), *t*(97) = 3.424, *p* = .001.

In PIMD individuals, this same visual preference was found in participant #2 (Nina), who showed significantly higher LT percentages on looked-at objects than on non-looked-at ones (74.38 vs. 25.62%, *SD* = 35.34), *t*(16) = 2.845, *p* = .012. Despite non-significant differences, four individuals showed the same tendency as TD children to look slightly longer at objects of joint attention: participants #1 (Zoe), #4 (Jane), #6 (Elise), and #7 (Mike). In contrast, we observed for the first time an opposite visual preference in one individual. Participant #5 (Betty) exhibited significantly higher LT percentages (averaged over only three trials) on non-looked at objects than on those reflecting RJA behaviors (89.67 vs. 10.33%, *SD* = 14.8), *t*(2) = -4.641, *p* = .043. This same pattern was also found in the LT percentages of participants #8 (John) and #9 (Tim). In addition, the data from the only four valid trials of participant #3 (Suzan) turned out to be problematic as zero visit durations were recorded on the AOI drawn around the looked-at object, thus making it impossible to calculate LT percentages for this participant. Results are detailed in Table 9.

**Table 9:** Mean (and *SD*) looking times (raw and percentage) at each of the two stimuli of the RJA-Task (i.e., looked-at vs. non-looked-at objects), consisting of four 15-second trials, of the control group and the nine PIMD participants.

	*n valid trials*	Raw looking times (LT) in seconds				LT in proportion (percentage)				Difference in LT percentages	
		Looked-at objects (RJA behaviors)		Non-looked-at objects		Looked-at objects (RJA behaviors)		Non-looked-at objects			
		*M*	*(SD)*	*M*	*(SD)*	*M*	*(SD)*	*M*	*(SD)*	*t*(n-1)	*p*
Control group (*n* = 27^1^)	97	1.847	*(1*.*208)*	1.424	*(1*.*139)*	57.98	*(22*.*96)*	42.02	*(22*.*96)*	3.424	.001
Participant #											
# 1 Zoe	18	0.498	*(0*.*467)*	0.504	*(0*.*435)*	53.47	*(29*.*86)*	46.53	*(29*.*86)*	0.493	.628
# 2 Nina	17	0.787	*(1*.*173)*	0.294	*(0*.*634)*	74.38	*(35*.*34)*	25.62	*(35*.*34)*	2.845	.012
# 3 Suzan	4	0		0.056	*(0*.*096)*						
# 4 Jane	8	0.229	*(0*.*285)*	0.143	*(0*.*204)*	50.72	*(44*.*0)*	49.28	*(44*.*0)*	0.046	.964
# 5 Betty	3	0.019	*(0*.*017)*	0.314	*(0*.*479)*	10.33	*(14*.*80)*	89.67	*(14*.*80)*	-4.641	.043
# 6 Elise	6	0.147	*(0*.*219)*	0.099	*(0*.*172)*	54.45	*(47*.*26)*	45.55	*(47*.*26)*	0.231	.827
# 7 Mike	23	0.956	*(1*.*064)*	0.605	*(0*.*636)*	57.39	*(34*.*61)*˟	42.61	*(34*.*61)*˟	1.024	.317
# 8 John	11	0.229	*(0*.*354)*	0.312	*(0*.*381)*	43.80	*(50*.*45)*	56.20	*(50*.*45)*	-0.407	.692
# 9 Tim	4	0.408	*(0*.*700)*	0.326	*(0*.*592)*	38.61	*(48*.*31)*	61.39	*(48*.*31)*	-0.471	.670

^***1***^. Data from five TD children were excluded from the analyses; the remaining sample consisted of 27 TD children (11 girls, 16 boys) aged 12 to 36 months (*M* = 22.8, *SD* = 7.3).

^˟^ Since the distribution of these variables was not normal (Shapiro-Wilk W-test = 0.852, *p* = .003), a non-parametric Wilcoxon matched-pair test was performed to compare them, which also turned out to be non-significant (Z = 1.08, *p* = .28).

#### Prosocial vs. antisocial behaviors and scenes

Focusing first on the final visual preference scene (cf. Table 10), we observed that the control group did not show a visual preference for either the pro-social or the anti-social puppet. Although TD children tended to look slightly longer at the hindering puppet, which had previously impeded the climber’s action, than at the helper (52.96 vs. 47.04%, *SD* = 14.73), this difference was not significant (*t*(43) = -1.335, *p* = .189). Similarly, the difference between the LT percentages for the two puppets was not significant for any of the PIMD individuals. However, the majority (five participants) also tended to look slightly longer at the anti-social puppet, while only four of them showed the opposite pattern.

**Table 10:** Mean (and *SD*) looking times (raw and percentage) at each of the two stimuli of the SME-Task’s final preference scene (i.e., prosocial—Or helper—Puppet vs. antisocial—Or hinderer—Puppet), consisting of two 15-second trials, of the control group and the nine PIMD participants.

	*n valid trials*	Raw looking times (LT) in seconds				LT in proportion (percentage)				Difference in LT percentages	
		Prosocial puppet		Antisocial puppet		Prosocial puppet		Antisocial puppet			
		*M*	*(SD)*	*M*	*(SD)*	*M*	*(SD)*	*M*	*(SD)*	*t*(n-1)	*p*
Control group (*n* = 29^1^)	44	4.829	*(1*.*911)*	5.426	*(2*.*061)*	47.04	*(14*.*73)*	52.96	*(14*.*73)*	-1.335	.189
Participant #											
# 1 Zoe	16	3.774	*(2*.*382)*	3.184	*(1*.*799)*	51.89	*(23*.*45)*	48.11	*(23*.*45)*	0.322	.752
# 2 Nina	14	5.180	*(3*.*109)*	4.777	*(3*.*146)*	49.72	*(29*.*33)*	50.28	*(29*.*33)*	-0.035	.972
# 3 Suzan	7	1.033	*(1*.*081)*	3.844	*(3*.*902)*	31.05	*(33*.*16)*	68.95	*(33*.*16)*	-1.511	.181
# 4 Jane	4	5.437	*(4*.*162)*	3.862	*(4*.*118)*	60.51	*(43*.*58)*	39.49	*(43*.*58)*	0.482	.663
# 5 Betty	4	6.606	*(2*.*906)*	3.454	*(3*.*020)*	66.0	*(26*.*7)*	34.0	*(26*.*7)*	1.199	.317
# 6 Elise	6	4.342	*(3*.*504)*	3.261	*(1*.*649)*	46.33	*(37*.*15)*	53.67	*(37*.*15)*	-0.242	.818
# 7 Mike	12	3.788	*(2*.*026)*	4.868	*(1*.*955)*	42.59	*(14*.*97)*	57.41	*(14*.*97)*	-1.714	.115
# 8 John	9	2.636	*(2*.*575)*	2.232	*(1*.*881)*	48.24	*(33*.*35)*	51.76	*(33*.*35)*	-0.158	.878
# 9 Tim	6	3.737	*(2*.*493)*	5.741	*(3*.*648)*	41.94	*(27*.*83)*	58.06	*(27*.*83)*	0.709	.510

^***1***^. Data from three TD children were excluded from the analyses; the remaining sample consisted of 29 TD children (12 girls, 17 boys) aged 12 to 36 months (*M* = 22.17, *SD* = 7.28).

Comparing the time spent gazing at each of the two climbing scenes (cf. Table 11), we then observed a significant difference in the control group who spent, on average, more time looking at the pro-social scene (86.37%, *SD* = 11.36) than the anti-social one (77.17%, *SD* = 13.91) (regardless of their order of presentation) (independent *t*-test: *t*(30) = 3.784, *p* < .001). Regarding the PIMD individuals, participant #7 (Mike) showed this same visual preference: on average, he spent significantly more time looking at the pro-social climbing scene (84.92%, *SD* = 14.61) than the anti-social one (67.03%, *SD* = 22.59), *t*(7) = 3.176, *p* = .016. Moreover, this same tendency was also found in six other individuals (although the differences were not significant). Only participants #5 (Betty) and #8 (John) showed the opposite pattern, resulting in higher LT percentages on the anti-social climbing scene.

**Table 11:** Mean (and *SD*) looking times (raw and percentage) at each of the two climbing scenes of the SME-Task (i.e., prosocial vs. antisocial scenes) each lasting 15 seconds, of the control group and the nine PIMD participants. Differences in LT percentages were tested using independent *t*-tests.

	*n valid trials*	Raw looking times (LT) in seconds					LT in proportion (percentage)^1^				Difference in LT percentages	
		Prosocial scene		Antisocial scene			Prosocial scene		Antisocial scene			
		*M*	*(SD)*	*M*	*(SD)*		*M*	*(SD)*	*M*	*(SD)*	*t*(n-1)	*p*
Control group (*n* = 31^2^)	31	15.978	*(2*.*101)*	14.277		*(2*.*573)*	86.37	*(11*.*36)*	77.17	*(13*.*91)*	3.784	.001
Participant #												
# 1 Zoe	8	12.024	*(3*.*113)*	10.875		*(3*.*444)*	70.73	*(18*.*31)*	63.97	*(20*.*26)*	0.674	.522
# 2 Nina	8	13.050	*(2*.*514)*	10.758		*(3*.*822)*	76.77	*(14*.*79)*	63.28	*(22*.*48)*	1.750	.124
# 3 Suzan	5	8.958	*(6*.*341)*	6.187		*(5*.*209)*	49.77	*(35*.*23)*	34.37	*(28*.*94)*	2.059	.109
# 4 Jane	5	8.895	*(6*.*861)*	6.266		*(5*.*946)*	52.32	*(40*.*36)*	36.86	*(34*.*98)*	1.450	.221
# 5 Betty	5	5.494	*(3*.*621)*	7.695		*(6*.*268)*	32.32	*(21*.*30)*	45.26	*(36*.*87)*	-0.577	.595
# 6 Elise	5	7.480	*(5*.*446)*	4.141		*(6*.*300)*	44.00	*(32*.*04)*	24.36	*(37*.*06)*	1.611	.182
# 7 Mike	8	15.286	*(2*.*630)*	12.066		*(4*.*066)*	84.92	*(14*.*61)*	67.03	*(22*.*59)*	3.176	.016
# 8 John	5	8.731	*(1*.*994)*	9.522		*(3*.*360)*	51.36	*(11*.*73)*	56.01	*(19*.*76)*	-0.556	.608
# 9 Tim	5	8.776	*(6*.*871)*	6.952		*(7*.*094)*	48.75	*(38*.*17)*	38.62	*(39*.*41)*	1.709	.163

^***1***^. Calculated as the time spent looking at each climbing scene divided by the duration of the scene.

^***2***^. Data from one TD child were excluded from the analyses; the remaining sample consisted of 31 TD children (13 girls, 18 boys) aged 12 to 36 months (*M* = 22.10, *SD* = 7.16).

## Discussion

### Feasibility of the eye-tracking-based experimental paradigm

Overall, findings appeared to support the feasibility of the eye-tracking-based experimental paradigm proposed in the present study for assessing individuals with PIMD. This was first shown through the preliminary analyses conducted on the gaze rates of PIMD participants. From the very first session, seven individuals spent most of the time of the session duration looking at the screen rather than elsewhere. This suggested that the duration and content of the paradigm were quite appropriate to their attentional resources. However, by repeating the procedure, we observed that these individual gaze rates were poorly consistent and tended to vary (more or less strongly, depending on the participant) from one session to another. Such results were nevertheless expected. Indeed, this intra-individual variability has already been highlighted by numerous studies conducted in the field of severe and multiple disabilities research [5, 10, 66]. Potential causes of this wide variability include the various neurological impairments of each PIMD individual but also the somatic factors (discomfort, pain, etc.) they each may have experienced at the time they were performing an experimental session. Indeed, the lack of functional language that characterizes the PIMD population prevents these individuals from being able to communicate their desires and/or needs [6], especially during daily activities, and consequently when they were performing a session in the framework of the present study. However, in order to limit the impact of possible somatic factors, we took a certain number of precautions beforehand. First, we decided to conduct repeated sessions (with a fixed minimum number of five) with each participant, alternating days of the week and times of the day (morning/afternoon). In addition, the experimenter systematically asked the support persons if the participant’s state of health and alertness were suitable for performing a session.

Since we assumed that the more sessions a participant did, the more reliable the results would be [5], we conducted up to eight sessions with three participants who were the most available (i.e., those with the fewest care visits scheduled and thus with the most free time). Nevertheless, when we compared the mean LT percentage of these participants obtained in their first five sessions with the percentage they actually obtained after completing their eight sessions, we found very similar values: 57.4 vs. 54.15% respectively for participant #1 (Zoe), 70.02 vs. 70.28% for participant #2 (Nina), and 68.48 vs. 71.23% for participant #7 (Mike). One possible interpretation of these results would be to consider that five repetitions of the same experimental procedure may be sufficient, at least in these three specific individuals, to account for intra-individual variability. Moreover, this could probably even be generalized to other participants because, in most cases, the standard deviations of participants who only completed five sessions were quite comparable to those of these three individuals who did eight. Furthermore, the comparison of the LT percentages of the PIMD individuals with those of the control group also seemed to be consistent with these initial findings. Indeed, a similar variance was found in the mean LT percentage of the 32 TD children in the control group, thus supporting the idea that five repetitions may be sufficient to reflect a certain variability expected at an estimated equivalent developmental level.

We then focused on the composition of the experimental phase of a session (i.e., six ‘blocks’, each lasting just under a minute, presented successively in random order) by investigating whether there were any order and/or fatigue effects that influenced the individual LT percentages. Again, the results seemed to support the feasibility of the paradigm, since the main effect of the order of presentation of the six blocks turned out to be significant only in one PIMD individual. This isolated result differed from our predictions since we had hypothesized that each block should be explored for equivalent durations, and thus not have any significant order effect. Similarly, we expected not to observe any fatigue effect, which was investigated by comparing the mean LT percentages on the first two blocks presented with the mean LT percentages on the last two blocks. This specific analysis was of greater importance because the presence of such a fatigue effect would imply an experimental phase that was too long for the participant’s attentional abilities, and consequently a poor feasibility of our paradigm. Therefore, the fact that this effect was indeed non-significant in all nine participants definitely supported the feasibility of the paradigm.

However, compared to the other PIMD individuals, participant #5 (Betty) showed somewhat abnormal recurrent results: indeed, through the five sessions she completed, it appeared that her LT percentage tended to increase as the experimental phase progressed. It is important to underline that Betty’s data set actually stood out from other individuals. Interestingly, she was the participant with the lowest mean LT percentage but also with the smallest standard deviation. In other words, Betty looked at the screen very little during her sessions and this was extremely stable across them. Based solely on her gaze rates, we would have tended to conclude that the proposed experimental paradigm was poorly suited to Betty’s attentional abilities. Nevertheless, the analysis of the symmetry of her visual exploration revealed some interesting additional results: despite the very small number of fixations (due to the very short visit durations), they were distributed over the entire surface of the screen, just like the visual exploration patterns observed in the other participants. Therefore, even though Betty spent little time looking at the screen, when she did look at it, she was able to scan its entire surface. This finally supported the feasibility of the paradigm for this participant, thus highlighting the possibility of collecting valid data in the visual preference tasks with her.

To conclude, we previously opened the Results section by pointing out that the control group looked significantly more at the screen than the PIMD individuals, which could have reflected a difference in the general level of attention between the two populations studied. However, the preliminary analyses discussed above clarify this result. Although TD children focused on the screen more than the PIMD participants, analyses of the latter’s gaze rates showed that there was no significant effect of block presentation order nor fatigue effect across the completion of experimental sessions. This suggests that the PIMD individuals tended to look at the screen intermittently throughout the session (and not in a decreasing manner from start to finish). This seems to indicate that their gaze was moving back and forth between the screen and ‘elsewhere’ (off-screen) more frequently than the control group, but then still managed to redirect their attention towards the screen during the experiment. Therefore, we can assume that we would not measure significantly higher gaze rates with a shorter assessment paradigm.

### Individual social-emotional abilities

The results of the analyses of visual preferences for the different tasks tested may, at first sight, seem inconclusive. Nonetheless, several relevant findings can still be highlighted from them.

First of all, one task stands out from the others by its particularly convincing results: visual scanning of facial features. Indeed, four PIMD individuals had similar results to the control group (i.e., significantly higher LT percentages at the eye area than at the mouth area). Moreover, this tendency to look longer at the eyes than the mouth was found in each of the nine PIMD participants. Thus, it is very interesting to observe that, despite their severe disabilities, all PIMD participants explored faces in a similar pattern to that already demonstrated in the TD population at different ages [46, 60]. This is probably related to the fact that, just like TD children, PIMD individuals are also highly exposed to human faces, especially in everyday tasks and daily living activities [1, 5]

Social orienting was another ability in which we also obtained significant results consistent with our predictions in the control group as well as in several PIMD participants. Indeed, this early ability of preferentially orienting to socially salient stimuli has already been observed in TD children aged between 22 and 51 months [32]. In line with these previous results, we found this preference in our control group of TD children aged, on average, 24 months. In addition, six PIMD participants showed the same tendency to look longer at the social scenes of the SO-Task, and in two of them the difference in LT percentages between the two stimuli (socially salient vs. non-social scenes) was also significant, just as for the control group. In addition, it should be noted that participant #9 (Tim) was among the three PIMD individuals who exhibited the opposite pattern (resulting in a tendency to look longer at non-social scenes). He presented autistic disorders and was first diagnosed with ASD at the age of 4. Given that the stimuli used in the present SO-Task had previously revealed significant differences in visual preferences between 20 children with ASD and a control group of 20 age-matched TD children (the latter preferring socially salient stimuli) in their original study [32], it is not surprising to find a similar reduced orienting to social scenes in Tim.

While the results of the control group on the RJA-Task were consistent with our expectations based on previous research (i.e., TD children did show a significant preference for objects looked at by the actress), this was undoubtedly the task in which PIMD participants performed the most trivially. Although one of them obtained a similar result to the control group, this was the only task where we found a significant preference for the stimulus opposite to our expectations (i.e., for the non-looked at objects) in one participant. Furthermore, this is the only task where no visit duration was recorded on either of the two compared AOIs, thus preventing the calculation of the proportion of LT in an individual. These results can partly be explained by several factors. Firstly, this is a more complex task than a ’standard’ visual preference procedure. Here, for a trial to be included in the analyses, the participant did not only have to explore the two AOIs compared (located on either side of the lower part of the screen), but also, and more importantly, he or she had to look at a third one: the face of the actress (located in the center of the upper part of the screen). These requirements generate three major difficulties related to (1) the asymmetric location of the three AOIs on the screen that required fine and precise scanning of the screen, and consequently high oculomotor control; (2) the size of the AOIs: the AOIs drawn here were considerably smaller than in the tasks where they each cover half of the screen and therefore required even more accurate oculomotor movements; and [3] the distinction of the two paired stimuli: although we selected only the video of the “intense surprise” condition of the original stimuli set [49], the two identical objects presented simultaneously could only be differentiated from each other on the basis of the direction of the actress’s gaze and by her expression of surprise. These two visual cues are extremely subtle, and thus potentially difficult to perceive. Moreover, these cues occurred in an AOI measuring only 450 x 350 pixels (i.e., the actress’s face): therefore, differentiating between them required much higher visual abilities, especially compared to other visual preference tasks where the two stimuli presented occupied half the screen each. These difficulties resulted in a considerable drop in the measured looking times, and consequently in the limited number of valid trials for each individual.

The decrease in looking times related to the AOIs’ size can similarly be seen in the outcomes of the face scanning task, where the eye and mouth areas were also delineated by very small AOIs. Although we have previously discussed the overwhelming evidence for this task, if we focus on the raw times, we can note that they are extremely low compared to the duration of the stimuli presentation. It is actually for this reason (i.e., insufficient data) that we did not formulate any hypotheses about potential interaction effects between the exploration of facial features and the emotional expression of the face, despite the fact that previous works have highlighted differences in the visual scanning of faces according to the emotions they express [60, 67]. Moreover, regarding emotion discrimination, the task was unexpectedly rather inconclusive in PIMD individuals where no significant difference was observed, whereas children in the control group exhibited a visual preference for angry faces. This skill has been little investigated in 2-year-old TD children before [68], thus it is interesting to find that they spent significantly more time looking at angry faces compared to happy ones. This same tendency was, nevertheless, also manifested by six PIMD individuals, representing more than half of them. One might assume that both TD infants and participants with PIMD are more exposed to smiling faces than to angry ones, which would indicate a reaction to novelty here [69].

Finally, the two remaining tasks did not reveal any significant results in the control group or in the PIMD individuals. Regarding firstly the visual processing of biological motion, we had hypothesized significant differences between the LT percentages on the two types of stimuli, specifically expecting to find a visual preference for biological motion. Such a result was not observed in any of the PIMD individuals, however it is comforting to note that neither did the TD children statistically look at one of the two stimuli longer than the other. This is again an interesting finding since the sensibility to biological motion has already been investigated in TD newborns [38] as well as in older children and in adults [70] but no study had previously explored this ability in infants of the age range of our control group.

Secondly, similar findings were observed regarding socio-moral evaluations. Nevertheless, their interpretation requires considering that the “Climbing the hill” paradigm combined with a visual preference procedure as used in the present study has previously been implemented only with 12 TD infants aged 3 months, where it revealed a preference for prosocial behaviors [71]. Indeed, although prosocial preferences have already been observed in older TD children (notably aged 24-36-month-olds), this has been done using different paradigms (e.g., “Playing a ball game”) and testing methods (e.g., manual choice) [72]. Moreover, some works have also observed opposite results: for example, by using the “Retrieving a dropped ball” paradigm, Hamlin (2014) found a preference (manifested through reaching behaviors) for anti-social behaviors in 27-month-old TD children [73]. Finally, the task used in the present study does not correspond to a ’standard’ visual preference procedure since it is actually directly related to the quality of the visual exploration of the two successive climbing scenes that preceded it. Therefore, we also decided to compare the time spent looking at each of these two climbing scenes to determine whether one of them had been gazed at longer than the other. This new analysis revealed a significant difference in the control group and in a PIMD individual: in both cases, the antisocial scene was gazed at statistically longer than the prosocial one. This finding could be explained by the fact that this scene would constitute a violation of one’s expectations, thus attracting more attention [50]. This pattern was also found in the majority (seven out of nine) of the PIMD participants, supporting this potential explanation.

### General conclusion

In conclusion, the results support the feasibility and the relevance of eye-tracking as a new theoretical and practical framework in the study of profound intellectual and multiple disabilities. Overall, individuals responded with good visual attention to the experimental content, resulting in high looking rates from the very first session performed that were not affected by the presentation of the tasks or their content. However, only a few individual patterns of visual preference were found to be similar to those of the control group, limiting the comparison between the two populations studied and therefore the interpretation of individual results.

The main limitation of this study concerns the method used. Indeed, the major limitation of the visual preference procedure is that when there is no statistically significant difference in the time participants spend looking at each of the two paired stimuli presented simultaneously, no conclusion can be drawn. Indeed, equivalent durations do not shed light on whether or not the person has perceived that they were different from each other because it is possible that the person can distinguish between them without showing a visual preference. This is, for example, what we observed with the results of the control group in the PL-task: although the TD infants were able to discriminate human movements from other non-biological motions for a long time (as early as a couple of days after birth), they did not exhibit any preference here. This limitation especially affected the interpretation of the results of the PIMD individuals, whose looking times were very rarely found to be significantly different. Therefore, using assessment instruments based solely on a visual preference procedure may suffer from a sensitivity limitation as they may not detect a skill that exists in an individual with PIMD.

Finally, an interesting perspective would be to develop computer-based trainings tailored on individual strengths and weaknesses to explore the learning potential abilities of these individuals. Because of the scarce significant results observed, we have, at this stage, only been able to establish the visual preference patterns of each individual in response to the different stimuli presented, whereas the implementation of such trainings would allow us to go much further by providing knowledge about the perceptual learning process of PIMD individuals. According to Gibson’s (1969) ecological approach to perception, perceptual learning is the means of discovering distinctive features and invariant properties of objects and events by extracting meaningful information from the environment to guide actions adaptively [74]. This specific and implicit type of early learning occurs at different levels of information processing. Distinguishing objects from their background, detecting their basic features (color, form, motion), location, or spatial relationships, as well as being able to identify and recognize them involve a wide range of cognitive functions (e.g., attention, visual discrimination, categorization) [75]. Hence, through daily exposure to repeated perceptual events associated with subjective experience, PIMD individuals may have developed unsuspected cognitive skills that cannot be assessed with existing evaluation methods and instruments but that eye-tracking-based training programs could help to identify, thus contributing to better understanding the perceptual learning process in this population. This would be particularly important for the support persons who work with individuals with PIMD, in whom progress may be slow and difficult to detect, and for whom delay of regression may be regarded as a positive outcome [2], providing them with directions to improve their assistance work.

## Supporting information

S1 Fig PL-Task stimuli representing biological (left) and non-biological (right) motion. The PL-Task consisted of two 20-second trials. Biological and non-biological motion were randomly assigned to the left or right sides of the screen for each trial.
(DOCX)

S2 Fig SO-Task stimuli (adapted from Franchini et al., 2016) representing socially salient (left) and non-social (right) scenes. The SO-Task was composed of eight 5-second trials consisting of four videos of a boy and four videos of a girl, both dancing solo (socially salient scenes) appearing twice on the left side of the screen and twice on the right side, next to moving geometric shapes (non-social scenes). Trials were presented in a random order.
(DOCX)

S3 Fig Happy (left) and angry (right) faces of reference F22 from the KDEF database (Lundqvist et al., 1998). To measure both visual scanning of facial features and discrimination of the emotions (happiness vs. anger), this pair of female emotional faces was presented during two 20-second trials with the two emotions laterally counterbalanced.
(DOCX)

S4 Fig Happy (left) and angry (right) faces of reference M17 from the KDEF database (Lundqvist et al., 1998). To measure both visual scanning of facial features and discrimination of the emotions (happiness vs. anger), this pair of male emotional faces was presented during two 20-second trials with the two emotions laterally counterbalanced.
(DOCX)

S5 Fig Areas of interest (AOIs) delineating happy (yellow) and angry (red) faces of reference F22 from the KDEF database (Lundqvist et al., 1998) and the facial areas of the eyes (blue) and mouth (green). Visual scanning of facial features was assessed by comparing the time spent looking at the eye vs. the mouth areas. Emotion discrimination was assessed by comparing the time spent looking at the happy vs. angry face.
(DOCX)

S6 Fig Areas of interest (AOIs) delineating happy (yellow) and angry (red) faces of reference M17 from the KDEF database (Lundqvist et al., 1998) and the facial areas of the eyes (blue) and mouth (green). Visual scanning of facial features was assessed by comparing the time spent looking at the eye vs. the mouth areas. Emotion discrimination was assessed by comparing the time spent looking at the happy vs. angry face.
(DOCX)

S7 Fig Example of RJA-Task stimuli (taken from Franchini et al., 2017) representing the actress directing her attention to one ("looked-at object", right) of the two identical moving objects in front of her. The RJA-Task was composed of four 15-second trials consisting of four videos in which an actress suddenly directed her attention to one of the two identical objects (arranged in front of her on either side of a table) by looking at it with an intensely surprised expression just after they started to move symmetrically. The side of the actress’ facial orientation (twice on the left and twice on the right), as well as the paired objects presented (ball, toy truck, plush rabbit, and flowers), were counterbalanced and the order of the four videos was randomized.
(DOCX)

S8 Fig Drawing representation of the different Areas of interest (AOIs) on the RJA-Task stimuli (taken from Franchini et al., 2017). The two AOIs delineating the object of joint attention ("looked-at object", right) and the "non-looked-at object" (left) are drawn in red. The additional AOI delineating the actress’ face, which had to be gazed for at least 300 ms for the trial to be validated, is drawn in blue.
(DOCX)

S9 Fig Example of SME-Task video sequences (adapted from the "climbing the hill" paradigm by Hamlin et al., 2007). (A) Screenshots of an example of the two consecutive climbing scenes representing prosocial (left) and antisocial (right) behaviors. The two behaviors occurred in a random order and the role of each puppet counterbalanced. The arrows depict the direction the puppets are moving. (B) Screenshot of the final visual preference scene representing both the "helper" (left) and the "hinderer" (right) puppets. The final scene consisted of two 15-second trials. Helper and hinderer were randomly assigned to the left or right sides of the screen for each trial.
(DOCX)

S1 Dataset Anonymized dataset including all the eye-tracking data (raw looking times and percentages) measured on the different individual trials of each experimental task in both the children of the control group and in the nine PIMD participants. (XLSX)
