# Supplementary material for: Eye-tracking-based experimental paradigm to assess social-emotional abilities in young individuals with profound intellectual and multiple disabilities
Source: PLoS One. 2022 Apr 14;17(4):e0266176. doi: 10.1371/journal.pone.0266176 (PMC9009637; doi:10.1371/journal.pone.0266176)
Supplement: S1 Fig — PL-Task stimuli representing biological (left) and non-biological (right) motion. The PL-Task consisted of two 20-second trials. Biological and non-biological motion were randomly assigned to the left or right sides of the screen for each trial. (DOCX) [file pone.0266176.s001.docx]

#
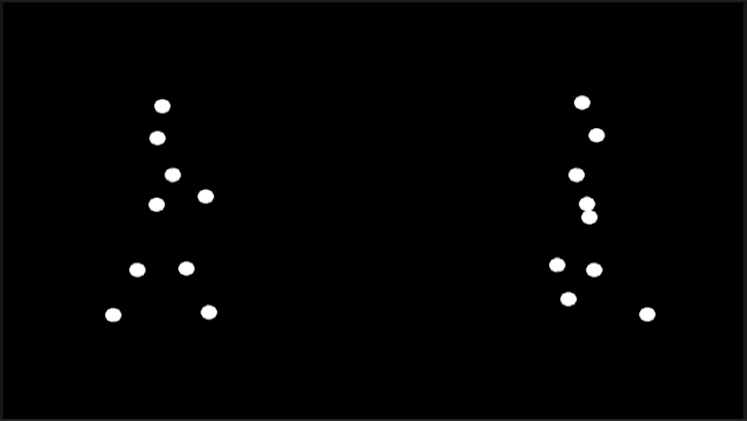


**S1 Fig. PL-Task stimuli representing biological (left) and non-biological (right) motion.** The PL-Task consisted of two 20-second trials. Biological and non-biological motion were randomly assigned to the left or right sides of the screen for each trial.
