# Supplementary material for: Eye-tracking-based experimental paradigm to assess social-emotional abilities in young individuals with profound intellectual and multiple disabilities
Source: PLoS One. 2022 Apr 14;17(4):e0266176. doi: 10.1371/journal.pone.0266176 (PMC9009637; doi:10.1371/journal.pone.0266176)
Supplement: S2 Fig — SO-Task stimuli (adapted from Franchini et al., 2016) representing socially salient (left) and non-social (right) scenes. The SO-Task was composed of eight 5-second trials consisting of four videos of a boy and four videos of a girl, both dancing solo (socially salient scenes) appearing twice on the left side of the screen and twice on the right side, next to moving geometric shapes (non-social scenes). Trials were presented in a random order. (DOCX) [file pone.0266176.s002.docx]

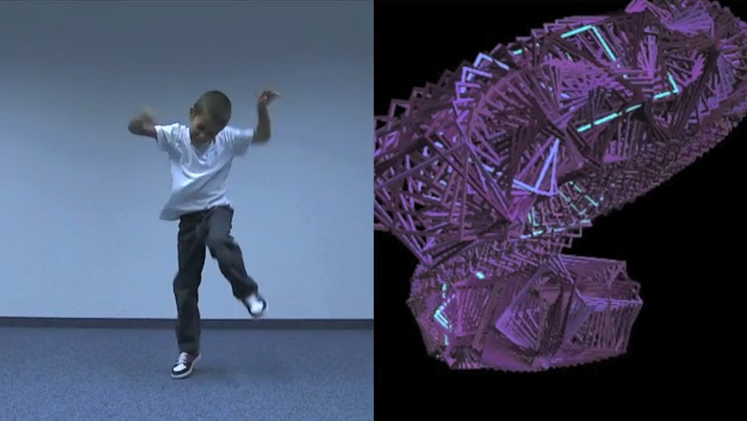


**S2 Fig. SO-Task stimuli (adapted from Franchini et al., 2016) representing socially salient (left) and non-social (right) scenes.** The SO-Task was composed of eight 5-second trials consisting of four videos of a boy and four videos of a girl, both dancing solo (socially salient scenes) appearing twice on the left side of the screen and twice on the right side, next to moving geometric shapes (non-social scenes). Trials were presented in a random order.
