# Supplementary material for: Eye-tracking-based experimental paradigm to assess social-emotional abilities in young individuals with profound intellectual and multiple disabilities
Source: PLoS One. 2022 Apr 14;17(4):e0266176. doi: 10.1371/journal.pone.0266176 (PMC9009637; doi:10.1371/journal.pone.0266176)
Supplement: S3 Fig — Happy (left) and angry (right) faces of reference F22 from the KDEF database (Lundqvist et al., 1998). To measure both visual scanning of facial features and discrimination of the emotions (happiness vs. anger), this pair of female emotional faces was presented during two 20-second trials with the two emotions laterally counterbalanced. (DOCX) [file pone.0266176.s003.docx]

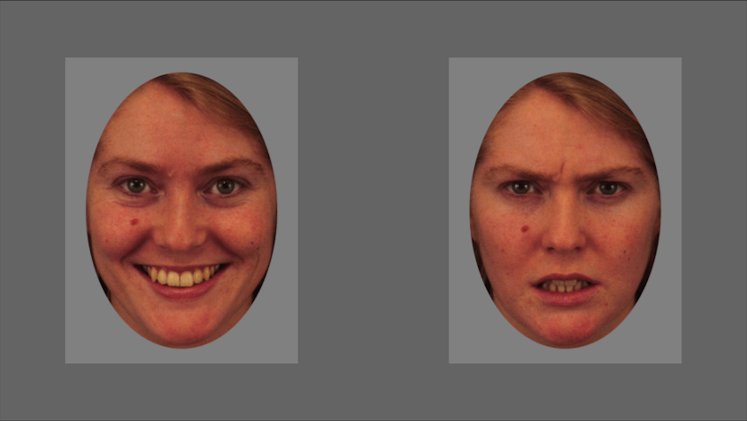


**S3 Fig. Happy (left) and angry (right) faces of reference F22 from the KDEF database (Lundqvist et al., 1998).** To measure both visual scanning of facial features and discrimination of the emotions (happiness vs. anger), this pair of female emotional faces was presented during two 20-second trials with the two emotions laterally counterbalanced.
