# Supplementary material for: Eye-tracking-based experimental paradigm to assess social-emotional abilities in young individuals with profound intellectual and multiple disabilities
Source: PLoS One. 2022 Apr 14;17(4):e0266176. doi: 10.1371/journal.pone.0266176 (PMC9009637; doi:10.1371/journal.pone.0266176)
Supplement: S6 Fig — Visual scanning of facial features was assessed by comparing the time spent looking at the eye vs. the mouth areas. Emotion discrimination was assessed by comparing the time spent looking at the happy vs. angry face. (DOCX) [file pone.0266176.s006.docx]

**
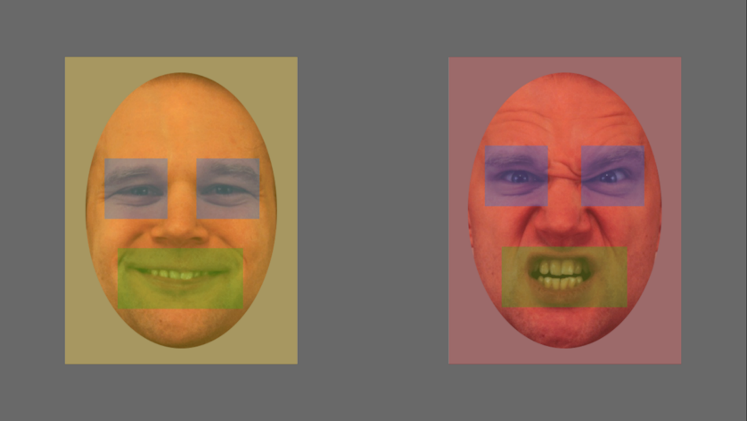
**

**S6 Fig. Areas of interest (AOIs) delineating happy (yellow) and angry (red) faces of reference M17 from the KDEF database (Lundqvist et al., 1998) and the facial areas of the eyes (blue) and mouth (green).** Visual scanning of facial features was assessed by comparing the time spent looking at the eye vs. the mouth areas. Emotion discrimination was assessed by comparing the time spent looking at the happy vs. angry face.
