# Supplementary material for: Eye-tracking-based experimental paradigm to assess social-emotional abilities in young individuals with profound intellectual and multiple disabilities
Source: PLoS One. 2022 Apr 14;17(4):e0266176. doi: 10.1371/journal.pone.0266176 (PMC9009637; doi:10.1371/journal.pone.0266176)
Supplement: S7 Fig — The RJA-Task was composed of four 15-second trials consisting of four videos in which an actress suddenly directed her attention to one of the two identical objects (arranged in front of her on either side of a table) by looking at it with an intensely surprised expression just after they started to move symmetrically. The side of the actress’ facial orientation (twice on the left and twice on the right), as well as the paired objects presented (ball, toy truck, plush rabbit, and flowers), were counterbalanced and the order of the four videos was randomized. (DOCX) [file pone.0266176.s007.docx]

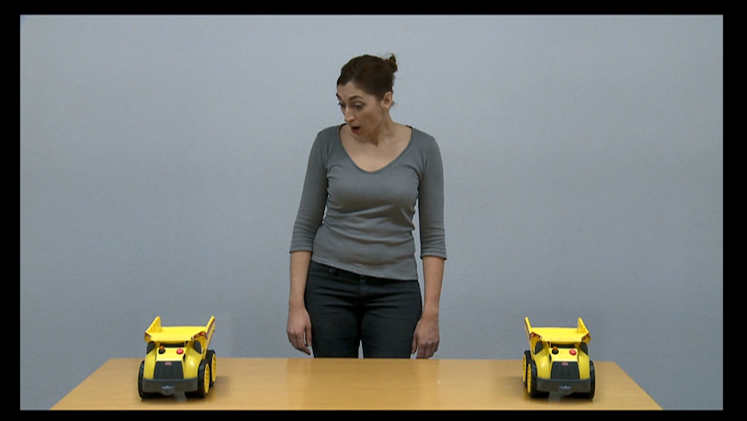


**S7 Fig. Example of RJA-Task stimuli (taken from Franchini et al., 2017) representing the actress directing her attention to one ("looked-at object", right) of the two identical moving objects in front of her.** The RJA-Task was composed of four 15-second trials consisting of four videos in which an actress suddenly directed her attention to one of the two identical objects (arranged in front of her on either side of a table) by looking at it with an intensely surprised expression just after they started to move symmetrically. The side of the actress’ facial orientation (twice on the left and twice on the right), as well as the paired objects presented (ball, toy truck, plush rabbit, and flowers), were counterbalanced and the order of the four videos was randomized.
