# Supplementary material for: Eye-tracking-based experimental paradigm to assess social-emotional abilities in young individuals with profound intellectual and multiple disabilities
Source: PLoS One. 2022 Apr 14;17(4):e0266176. doi: 10.1371/journal.pone.0266176 (PMC9009637; doi:10.1371/journal.pone.0266176)
Supplement: S8 Fig — The two AOIs delineating the object of joint attention ("looked-at object", right) and the "non-looked-at object" (left) are drawn in red. The additional AOI delineating the actress’ face, which had to be gazed for at least 300 ms for the trial to be validated, is drawn in blue. (DOCX) [file pone.0266176.s008.docx]

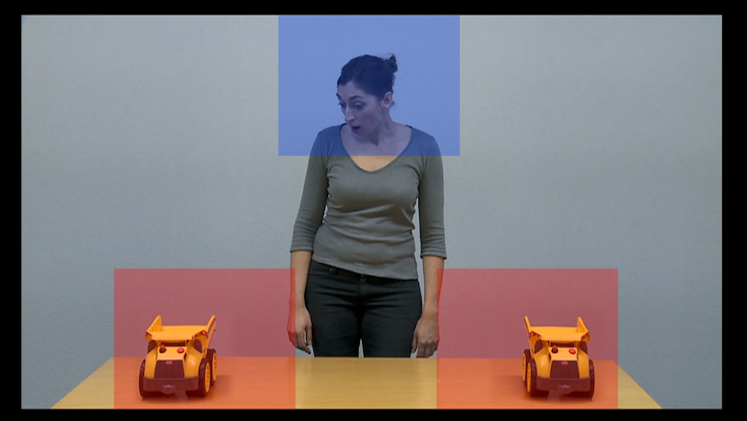


**S8 Fig. Drawing representation of the different Areas of interest (AOIs) on the RJA-Task stimuli (taken from Franchini et al., 2017).** The two AOIs delineating the object of joint attention ("looked-at object", right) and the "non-looked-at object" (left) are drawn in red. The additional AOI delineating the actress' face, which had to be gazed for at least 300 ms for the trial to be validated, is drawn in blue.
