# Supplementary material for: Eye-tracking-based experimental paradigm to assess social-emotional abilities in young individuals with profound intellectual and multiple disabilities
Source: PLoS One. 2022 Apr 14;17(4):e0266176. doi: 10.1371/journal.pone.0266176 (PMC9009637; doi:10.1371/journal.pone.0266176)
Supplement: S9 Fig — (A) Screenshots of an example of the two consecutive climbing scenes representing prosocial (left) and antisocial (right) behaviors. The two behaviors occurred in a random order and the role of each puppet counterbalanced. The arrows depict the direction the puppets are moving. (B) Screenshot of the final visual preference scene representing both the "helper" (left) and the "hinderer" (right) puppets. The final scene consisted of two 15-second trials. Helper and hinderer were randomly assigned to the left or right sides of the screen for each trial. (DOCX) [file pone.0266176.s009.docx]

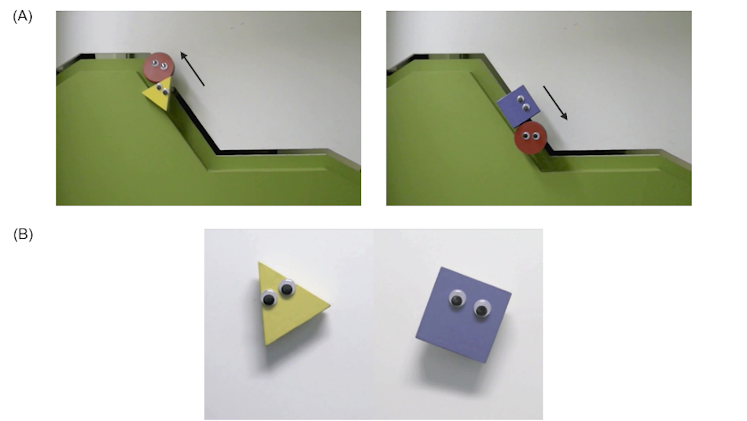


**S9 Fig. Example of SME-Task video sequences (adapted from the "climbing the hill" paradigm by Hamlin et al., 2007).** (A) Screenshots of an example of the two consecutive climbing scenes representing prosocial (left) and antisocial (right) behaviors. The two behaviors occurred in a random order and the role of each puppet counterbalanced. The arrows depict the direction the puppets are moving. (B) Screenshot of the final visual preference scene representing both the "helper" (left) and the "hinderer" (right) puppets. The final scene consisted of two 15-second trials. Helper and hinderer were randomly assigned to the left or right sides of the screen for each trial.
